# A *Staphylococcus aureus* Proteome Overview: Shared and Specific Proteins and Protein Complexes from Representative Strains of All Three Clades

**DOI:** 10.3390/proteomes4010008

**Published:** 2016-02-19

**Authors:** Chunguang Liang, Dominik Schaack, Mugdha Srivastava, Shishir K. Gupta, Edita Sarukhanyan, Anne Giese, Martin Pagels, Natalie Romanov, Jan Pané-Farré, Stephan Fuchs, Thomas Dandekar

**Affiliations:** 1 Department of Bioinformatics, Biocenter, University of Würzburg, 97074 Würzburg, Germany; liang@biozentrum.uni-wuerzburg.de (C.L.); dominik.schaack@uni-wuerzburg.de (D.S.); mugdha.srivastava@uni-wuerzburg.de (M.S.); shishir.gupta@uni-wuerzburg.de (S.K.G.); edita.sarukhanyan@uni-wuerzburg.de (E.S.); 2 Institut für Mikrobiologie Ernst-Moritz-Arndt-Universität Greifswald, Friedrich-Ludwig-Jahn-Straße 15, D-17487 Greifswald, Germany; anne.giese@uni-greifswald.de (A.G.); pagelsm@uni-greifswald.de (M.P.); janpf@uni-greifswald.de (J.P.-F.); 3 Structural and Computational Biology, European Molecular Biology Laboratory, Mayerhofstr. 1, D-69126 Heidelberg, Germany; natalie.romanov@embl.de; 4 FG13 Nosocomial Pathogens and Antibiotic Resistance, Robert Koch Institut (RKI); Burgstr. 37; D-38855 Wernigerode, Germany; FuchsS@rki.de

**Keywords:** *Staphylococcus aureus*, proteome, protein complexes, model strain

## Abstract

*Staphylococcus aureus* is an important model organism and pathogen. This *S. aureus* proteome overview details shared and specific proteins and selected virulence-relevant protein complexes from representative strains of all three major clades. To determine the strain distribution and major clades we used a refined strain comparison combining ribosomal RNA, MLST markers, and looking at highly-conserved regions shared between strains. This analysis shows three sub-clades (A–C) for *S. aureus*. As calculations are complex and strain annotation is quite time consuming we compare here key representatives of each clade with each other: model strains COL, USA300, Newman, and HG001 (clade A), model strain N315 and Mu50 (clade B) and ED133 and MRSA252 (clade C). We look at these individual proteomes and compare them to a background of 64 *S. aureus* strains. There are overall 13,284 *S. aureus* proteins not part of the core proteome which are involved in different strain-specific or more general complexes requiring detailed annotation and new experimental data to be accurately delineated. By comparison of the eight representative strains, we identify strain-specific proteins (e.g., 18 in COL, 105 in N315 and 44 in Newman) that characterize each strain and analyze pathogenicity islands if they contain such strain-specific proteins. We identify strain-specific protein repertoires involved in virulence, in cell wall metabolism, and phosphorylation. Finally we compare and analyze protein complexes conserved and well-characterized among *S. aureus* (a total of 103 complexes), as well as predict and analyze several individual protein complexes, including structure modeling in the three clades.

## 1. Introduction

Systems biology provides an integrated view on bacterial adaptation under changing environmental conditions, including its metabolism, its transcriptome, and proteome [1]. Furthermore, protein complexes have already been the topic of several studies; for instance, in *E. coli* (EcoCyc has a useful dataset on protein complexes [2]), and there are always new examples on protein complexes analyzed in *E. coli* [3] and in other prokaryotes (reviewed in [4]). However, not much is known about protein complexes and their specific components in *Staphylococcus aureus*.

Omics studies, in particular proteomics, are essential in understanding and revealing the life style of *S. aureus* [5,6]. *S. aureus* is a Gram-positive model organism and a challenging pathogen in clinical infections. It is not easy to establish a general overview on the *S. aureus* proteome and protein complexes: identification of conserved and strain-specific proteins requires all-against-all sequence comparisons; structure predictions require detailed calculations even for a single protein complex. Nevertheless, in order to have a good strain overview and look at representative proteins and protein complexes we first performed a refined strain comparison combining two well-established phylogenetic markers, *i.e.*, ribosomal RNA and MLST markers (including *arc*, *aroE*, *glpF*, *gmk*, *pta*, *tpi*, *yqiL*). For the best phylogenetic resolution we then considered highly-conserved regions shared between *S. aureus* strains. Based on this high-resolution analysis and considering the 64 *S. aureus* genomes completely known we can show that there are three sub-clades (A-C) encompassing all *S. aureus* strains and give a first view on the complete repertoire of proteins and complexes conserved among all these strains. In order to avoid both too complex calculations, and the annotation of all strains individually and completely for each protein, we next compare key representatives of each clade amongst each other: model strains COL, USA300, Newman, and HG001 (clade A), model strain N315 and Mu50 (clade B), and ED133 and MRSA252 (clade C). We establish strain-specific proteins that distinguish the different strains from each other and look at pathogenicity islands with a high number of strain-specific proteins. Next, we analyze important protein repertoires involved in virulence, cell wall component/glycosylation and look at individual strain-specific protein complexes in the three clades. For strain-specific protein complexes we give several detailed structure predictions. Furthermore, the sequence comparisons are complemented by predictions from bioinformatics using three different gene context methods, evidence from databases, co-expression, and text mining. We also indicate which of these interactions are of particular interest for further experimental investigation.

We find that there is surprisingly high diversity, complexity and adaptation potential of proteome and protein complexes amongst *S. aureus* strains. This highlights the need for detailed systems biological investigations and high-throughput experiments to better understand the suggested interactions and complexes as well as their intricate regulation. Several of these improve *S. aureus* adaptation and its challenging capacity for infection. As a first overview, our study shows which proteins and complexes are conserved among all three *S. aureus* clades and models strain-specific proteins and protein complexes from key representatives of each clade.

## 2. Materials and Methods

### 2.1. Genome-Based Comparisons

**Figure 1:**
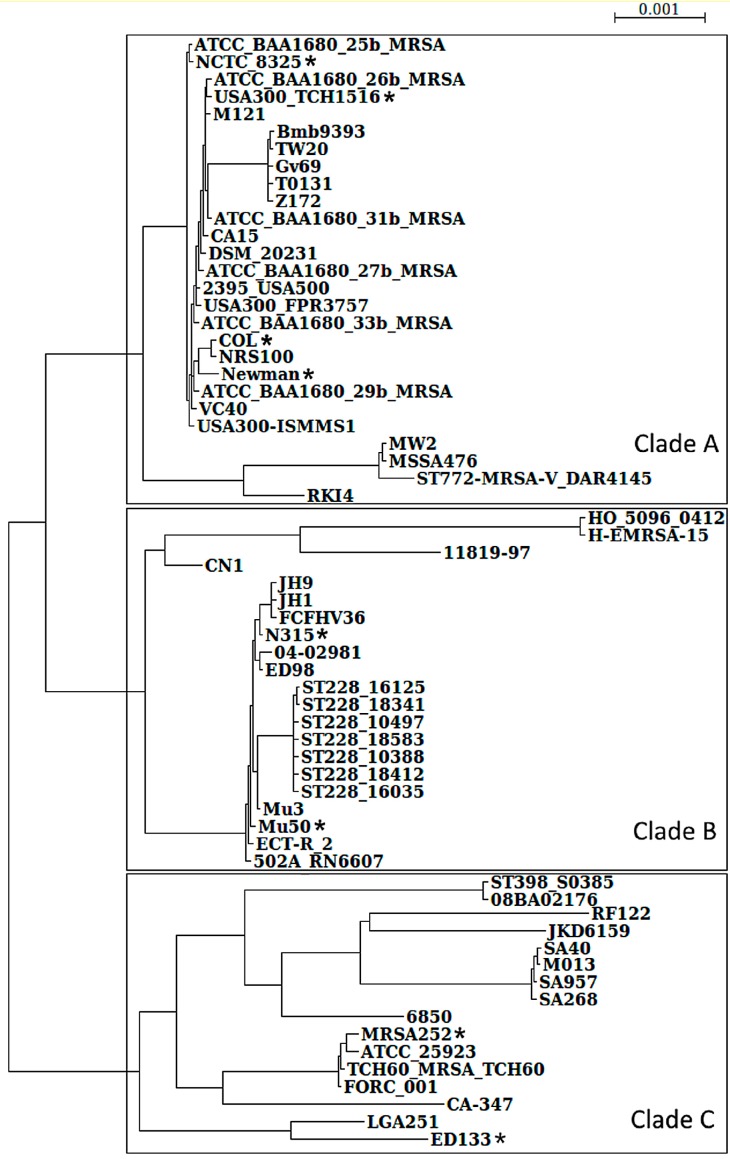
Phylogenetic analysis on 64 *Staphylococcus aureus* strains. All the completely sequenced *S. aureus* genomes were compared considering both MLST and 16S rRNA phylogeny; the generated maximum likelihood tree indicates *S. aureus* strains can be classified into three large clades. Asterisks indicate the positions of representative strains for the different clades: COL, NCTC8325 (with its derivative HG001), USA300, and Newman are all in the first clade A (top), whereas N315 and Mu50 are in the second clade B (middle). ED133 and MRSA252 were chosen as representative strains for clade C (bottom).

A systematic genome comparison included 64 genomes (Figure 1; a detailed list with accession numbers in Supplementary File S1, Table S1) and applied BLAST+ (version 2.2.31) [7] for identifying orthologous and non-orthologous proteins, core genome, and accessory genomes. Orthology was determined *in silico* by sufficient identity of amino acids (>50%) and respective coverage (the shorter partner covered 75% of the partner protein sequence and up to 125% for the longer partner). The reasoning here was that these high criteria for sequence identity and sequence coverage identify, in most cases, true orthologs and, in particular, functional identical proteins in the two compared strains. In addition, local synteny was considered to determine all the *S. aureus* core genes. Non-coding genes (in particular RNA genes) were carefully excluded from this comparison as the proteome was analyzed.

### 2.2. Modeling Complexes

**Figure 2:**
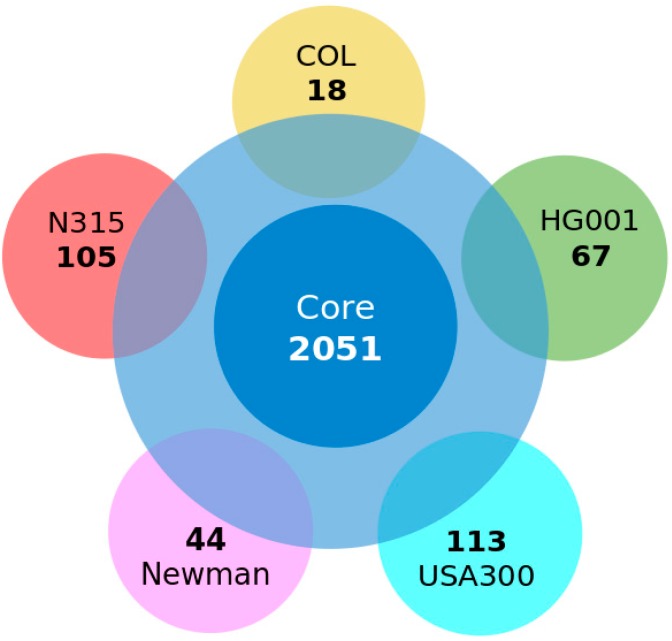
Sequence-based proteome comparison between representative *S. aureus* strains from clade A and clade B against the background of all strains. Blue indicates the calculated *S. aureus* core proteome (core CDS) of *S. aureus* of 2051 proteins after comparing proteome data predicted from the 64 completely sequenced *S.* *aureus* genomes. However, there are 2598 CDS (light blue) shared among COL, N315, Newman, HG001 (NCTC8325), and USA300 (USA300_TCH1516). There are 18 strain-specific genes present only in COL (yellow), 105 strain-specific genes in N315 (salmon), 67 in HG001, 44 in Newman (magenta), and 113 in USA300 (cyan).

By sequence comparisons, we first identified the conserved complexes which formed the core-complexome, noting strain-specific variation, as well as strain-specific additions. The total number of proteins in *S. aureus* strains is high (Figure 2; 13,284 *S. aureus* proteins, partly participating in various complexes). However, the total number of known, well-annotated, and conserved protein complexes over all compared *S. aureus* strains is lower (Figure 2; 103 complexes). For this result, knowledge and experimental data about firmly-established complexes is combined with the number of conserved proteins calculated from the all-against-all comparison. Hence, using data on studies of protein complexes in Gram-positive bacteria, such as *M. pneumoniae* [8], and our own studies on protein complexes in *S. aureus* and related organisms [1,4], we derive the list of known and established protein complexes which are conserved in *S. aureus* (see Supplementary Table S2). For comparison, we calculated the total number of proteins which all, potentially, can be involved in protein complexes (“other proteins and protein complexes”). Note, again, that confirming the presence of a complex can only be concluded from experimental data, and that the information whether it is conserved between strains requires not only numerous sequence comparisons (all-against-all proteome comparisons) but also proper annotation of the reading frames in all strains included in the comparison. Furthermore, for three-dimensional data on the complex it is, in addition, necessary to have a structure template. This is often available for central parts of the complexes, but in many cases for additional proteins no structure template for homology modeling and calculating the three-dimensional coordinates was not available. Hence, we had to be, and are, quite selective in our detailed protein structure comparisons.

Next, the type of interaction was predicted using data from the STRING database [9], as well as our own algorithms (see below: AnDOM structure prediction [10] and GoSynthetic database [11] to check for gene context (co-occurrence, fusion, neighbourhood), direct physical interaction according to databases, to homologues, and according to gene expression data [12,13].

### 2.3. Structure Annotation with AnDOM

The original AnDOM (annotation of structural domains) database was updated to the most recent version of SCOP (1.75 release) [14]. Furthermore, access to updated BLAST and BLAST+ packages [7] was added to the AnDOM tool. Matrix profiles were calculated for each of the specifically-generated structural related sequence alignments (via SCOP). The IMPALA package [15] was used to obtain position specific scoring matrices (PSSMs) from the PSI-BLAST outputs. Source code, aravind105 database, and wolf1187 database are available and were downloaded from the NCBI ftp site [16]. IMPALA employs a more refined analysis of statistical significance and, unlike PSI-BLAST or BLAST, guarantees the optimal local alignment with its implemented pairwise comparison algorithm, such as the rigorous Smith–Waterman algorithm. [17]. A detailed description of the methodology used in the initial AnDOM project is available [10]: briefly, a curated set of high-resolution domain structures is used to identify those regions in the protein which are homologous to one of the structure domains stored in the database. These regions are highlighted and identified in the protein sequence together with a pointer to the three-dimensional coordinates (PDB structure) of the sequence. However, we utilize here over 13 years of accumulated structural knowledge, as made available in the SCOP database (38,211 entries in release 1.75 from June 2009 covering 110,800 domains compared to 3,179 protein domains grouped in 498 families and 366 super-families in the release from 1995). Moreover, PSSMs were based on the wealth of sequence information that also has accumulated (55,270,679 sequences in UniProtKB/TrEMBL 2015 (release 2015_12) compared to 146,720 sequences in UniProtKB/Swiss-Prot major release 43.0 in March 2004). Highly-sensitive HMM predictions [17] extended our structure prediction alignments further.

### 2.4. Phylogenetic Analysis

We wanted an overview on all complexes in *S. aureus*. This is a huge task; hence, we were only able to focus on well-characterized strains representative of the three phylogenetic clades of *S. aureus*. We, thus, give only an overview on the protein inventory of these strains and point out specific protein complexes. Pictures focus on those few proteins whose structure information and modeling templates are available. The different clades are only visible if, first, a phylogenetic analysis is done. Phylogenetic analysis was performed on the genome data of 64 completely-sequenced *S. aureus* strains. MLST markers (*arc*, *aroE*, *glpF*, *gmk*, *pta*, *tpi* and *yqiL*) and highly-conserved regions shared between strains are reported here for the first time, as well as 16S rRNA genes that were selected for global alignment. The generated profile was further analyzed in PhyML [18] to obtain a maximum-likelihood tree. Next, from this overview three clades, A, B and C, became apparent. From this representative model strains were chosen for further detailed analysis. The representative strains were picked according to the criteria to have a well-annotated genome as they are widely studied in the scientific community and having a phylogenetic position in the respective clade. We, thus, considered *S. aureus* COL, USA300, Newman, HG001, N315, Mu50, ED133, and MRSA252 (Supplementary Table S1 gives details, including clades, on these strains).

## 3. Results

### 3.1. S. aureus Strains Form Three Clades

To have a good overview on all *S. aureus* strains requires all-against-all protein sequence comparisons of all strains. This is very time consuming and, hence, we restricted this effort to all *S. aureus* strains with a completely known genome, as well as good annotation. Furthermore, we wanted to solidly establish the number of sub-clades involving these 64 strains. To achieve this, we first considered data from two well-established phylogenetic markers, *i.e.*, ribosomal RNA trees, as well as MLST marker trees (comparing *arc*, *aroE*, *glpF*, *gmk*, *pta*, *tpi*, *yqiL*). For the best phylogenetic resolution we took highly-conserved regions into account that are shared between most *S. aureus* strains as well as concatenated established maker genes (*arc*, *aroE*, *glpF*, *gmk*, *pta*, *tpi*, *yqiL*) and the information from 16S ribosomal RNA. Taken together, this offers us an improved view on their phylogenetic relationship and leads to the phylogenetic tree shown in Figure 1.

Three major clades formed from the 64 *S. aureus* strains compared become readily apparent from this phylogenetic view. Note *S. aureus* MSHR1132 (Genbank accession number: FR821777) was excluded from this comparison, because it was recently reassigned to the new species *Staphylococcus* *argenteus* [19]. Figure 1 shows these three clades (A–C) for *S. aureus* (a detailed strain list for the three clades is found in Supplementary Table S1). We note that detailed structure predictions on protein complexes are time consuming and require available structure templates where the three-dimensional structure is known. Annotation of individual protein sequences requires detailed strain information and information on protein complexes requires available experimental data. Hence, for our more detailed analysis, we used only those *S. aureus* strains that are well-characterized representatives for the three clades (Figure 1). We see the general relatedness of four model strains (all in clade A), and the strong variation in USA300 (Figure 1): most of the well-known *S. aureus* model strains are situated in clade A. Here we looked at four model strains in detail: *S. aureus* COL, USA300, Newman, and HG001 (this is an *rsbU*-restored derivative of NCTC 8325). Interestingly, it turns out that several often-used strains in the A clade are clonal [20]. To acquire first insights into the not-so-well-characterized proteins and complexes from the two other clades we looked at the proteins and complexes of four further strains: for clade B this involved strains N315 and Mu50, and for clade C we considered ED133 and MRSA252.

### 3.2. Conserved Protein Complexes and Strain-Specific Proteins

**Figure 3:**
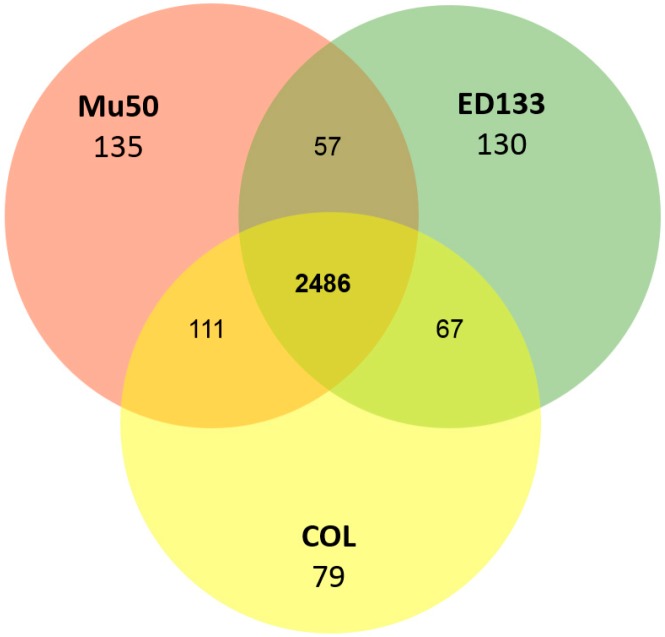
Proteome comparison between three representative *S. aureus* strains from the three major clades against the background of all strains. The detailed pair-wise comparison was performed among *S. aureus* COL (clade A), Mu50 (clade B), and ED133 (clade C). COL has 79 strain-specific genes (yellow) which are missing from ED133 and Mu50 strains. There are 135 strain-specific genes in Mu50 (salmon) absent in COL and ED133. In addition, 130 strain-specific genes can only be found in ED133 (green).

To gain further insight into the *S. aureus* proteome and to identify functional, as well as physical interactions that take place between proteins thereof, we compared five strains—COL, HG001, Newman, and USA300 (clade A)—to N315 (clade B), regarding their proteome (Figure 2) as predicted from the genome sequences to all 64 individual *S. aureus* strains (a detailed list on the 64 genomes compared including accession numbers for their proteome sequences and clade information is given in supplementary material Table S1). For the identification of protein complexes and the core proteome, we considered only core protein genes with a coding sequence (CDS) and did not consider RNA genes, since we studied here only the proteome. The central circle in Figure 2 indicates the core proteome building on the data from the *S. aureus* pan-genome, which includes all the latest 64 completely-sequenced genomes (Figure 2). For comparison, Figure 3 shows a proteome comparison between three representative *S. aureus* strains (COL, ED133 and Mu50) from the three major clades against the background of all strains in a full Venn diagram. Table 1 summarizes all eight strains looked at in detail (including MRSA252).

Next, our analysis established a detailed list of conserved protein complexes. Sequence analysis and all-against-all comparisons of the genome-encoded protein content of the 64 *S. aureus* strains helps to define the central core proteome. Furthermore, the strain-specific proteins in the model strains were determined. A strain-specific protein list from a complete all-against-all comparison of protein coding genes is, instead, less informative, as it increasingly minimizes strain-specific genes as more genomes are compared. The strain-specific complexes can be predicted with near certainty if we consider enzyme subunits, ABC transporters, and gene context methods, as well as a protein interaction database (see Materials and Methods, e.g. [8]). For less well-annotated proteins, and even more for presently uncharacterized protein complexes, the level of accuracy is lower, in particular with regard to the differentiation between direct physical interaction (common complex) and functional association (common pathway).

**Figure 4:**
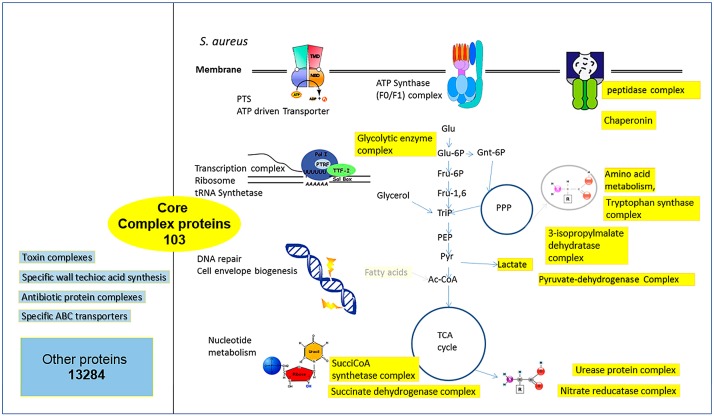
Conserved protein complexes and pathways in *S. aureus*. Strain variation of protein complexes in the analyzed strains leaves key metabolic functionalities unchanged (103 core complex proteins). Conserved protein complexes are highlighted in yellow. There are 13,284 other proteins (left, blue) which, to some extent, may be shared between several strains and form even protein complexes, but participation and size of these protein complexes varies highly and in a strain-specific way.

The list of the 103 conserved protein complexes is provided as supplementary material (Table S2). The type of interaction was predicted, using data from the STRING database [9] (version 10 from 2015). This database predicts protein-protein interactions based on different criteria: conserved gene neighborhood or fusion is considered first. Thus, if two genes occur next to each other in many prokaryotic genomes, it predicts that the encoded proteins also interact directly. It was shown that if conservation of gene neighborhood is observed in 100 or more genomes, this is a highly reliable indication for protein interaction (Bayesian probability for correct prediction around 0.99). Related criteria are gene fusion of the two protein genes observed in several genomes or the common presence or common absence in many genomes. In addition, the STRING database included large-scale protein-protein interaction data from experimental screens in model organisms (*E. coli*, yeast, human), and a huge number of gene expression datasets as co-expression of two proteins is another predictor that these two proteins interact. Finally, evidence from literature is considered; in particular, if two proteins are mentioned together in research articles. In addition we looked at protein homologies and information from proteins in related organisms. All of these indications for protein-protein interactions are integrated into a total Bayesian score for the probability that two proteins interact. We considered only highly reliable interaction predictions (Bayesian score from STRING tool at least 0.9; evaluation example in Supplementary File S1). Furthermore, we used our algorithms to check for gene context (co-occurrence, fusion, and neighborhood), direct physical interaction according to this, and other, databases, homology, and gene expression data. Finally, in order to further confirm complexes, where available, evidence from the literature was considered, as well as the established rule that enzyme subunits directly and physically interact to fulfil their job. These predicted interactions and involved pathways for *S. aureus* conserved protein complexes are summarized in Figure 4.

Protein complexes can, of course, be compared in the different clades focusing on functional categories and considering the well-annotated representative strains. Nevertheless, without a huge amount of strain-specific data we can only make predictions according to homology-based sequence comparisons and the results depend also on the strains compared against each other and their distribution among clades. Table 2 shows in section 2a the result for a clade A against clade B comparison regarding central cell wall modification and synthesis proteins. Complexes are indicated in color and, in 2b, we see the results for virulence factors (here, prediction of complexes is less clear) and now, looking at all three clades, comparing COL, Mu50, and ED133 as representative strains. Finally, Table 2c looks at phosphorylation by the *stk*/*stp* system which is easily identified and compared in the clade A and clade B strains. However, the *stk*/*stp* system regulates many other proteins and protein complexes, which each needing to be identified and monitored in individual strains. This has only recently been started, focusing on clade A strain *S. aureus* COL (see [21] for detailed data on phosphorylated proteins and protein complexes).

**Figure 5:**
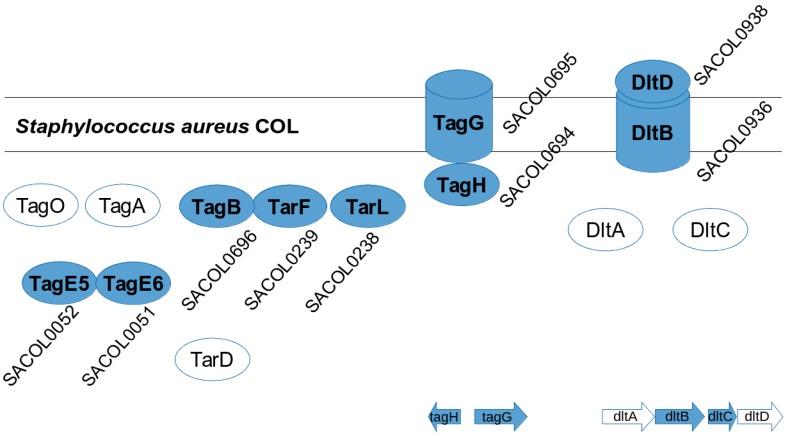
*S. aureus* COL protein complexes involved in wall teichioc acid biosynthesis. TagG and TagH form a tight protein complex playing a role in translocation, DltD and DltB form another protein complex responsible for alanine incorporation. Furthermore TagE5 and TagE6, encoded by SACOL0052 and 0051, are specific for *S. aureus* COL compared to the other strains. Moreover, DltC and DltA are as well as TagO, TagA, TagB, TarF and TarL and the specific complexes TagE5 and TagE6 (refinement) are all enzymes of cell wall synthesis, they form a sort of conveyor belt for cell wall synthesis and are all associated with each other (close enough to the membrane to be found in membrane preparations). Proteins forming complexes are noted as filled shapes, whereas others (unfilled circles) are not subunits of any protein complex, however, they are also involved the cell wall biosynthesis pathway. In the bottom, we sketch the cell wall operon structure. The list of strain-specific proteins and sequences is given in Supplementary Table S5.

**Figure 6:**
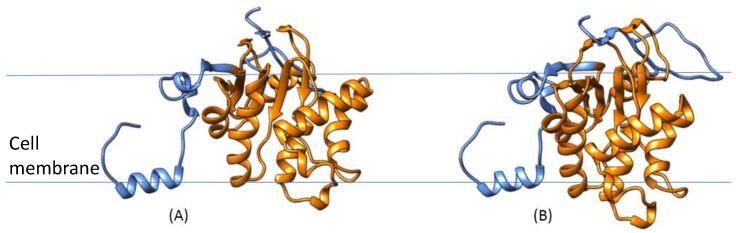
ABC transporter structure (SACOL0694). Shown in cartoon representation and indicating secondary structure types is the predicted protein structure according to our prediction tool AnDOM. The structure calculated covers the full sequence, and the putative ABC transporter TM0544 domain is shown in orange (Family c.37.1.12: ABC transporter ATPase domain-like). We give here views (**A**,**B**) from both sides of the protein (top and bottom). Blue lines indicate the borders of the cell membrane.

**Figure 7:**
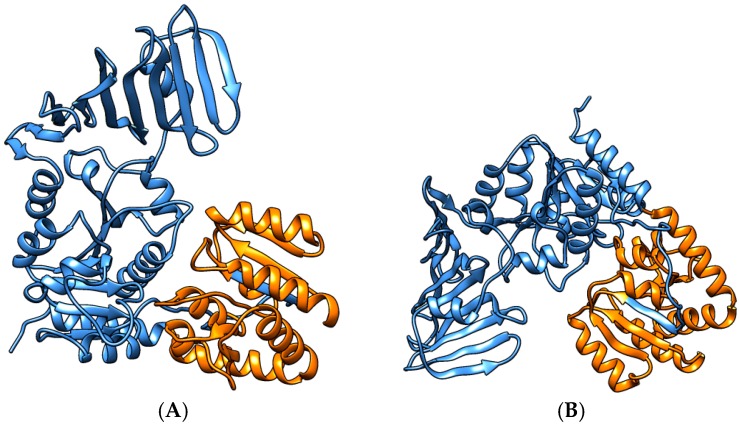
Lipopolysaccharide core biosynthesis protein (RfaG, SACOL0052). Shown in cartoon representation and indicating secondary structure types is the predicted protein structure according to our prediction tool AnDOM. The structure calculated covers the full sequence, and the putative lipopolysaccharide core biosynthesis protein RfaG domain is shown in orange color (Family c.87.1.8: glycosyltransferases group 1). Shown are the views on both sides of the protein (**A)** versus (**B**).

**Figure 8:**
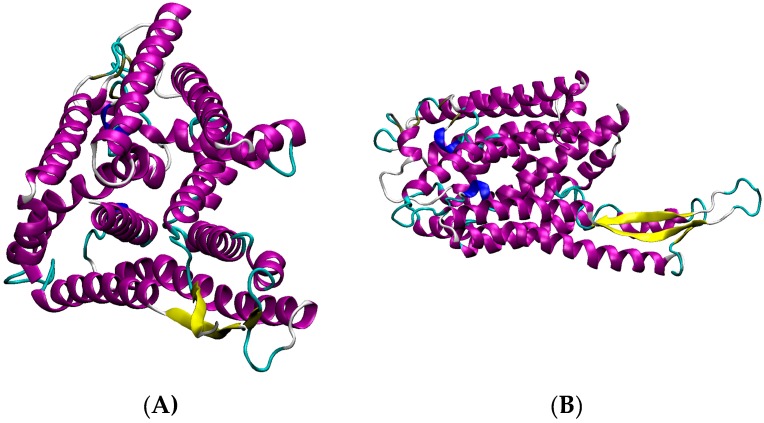
Different views of SecY of MRSA252. Cartoon representation of SecY secondary structure, helices in purple, turns in blue, and beta sheets in yellow. Two different perspectives are shown (starting view (**A**) and alternative perspective (**B**)).

**Figure 9:**
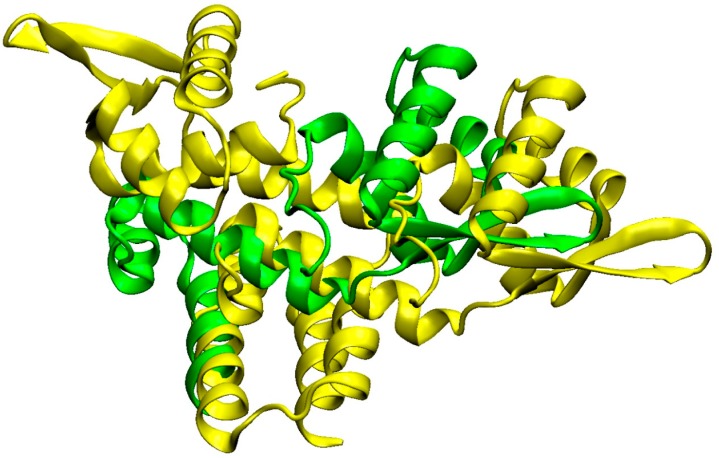
Structure comparison of mecA-type methicillin resistance repressor MecI protein. Shown is a superposition comparison between N315 (green) and Mu50 (yellow). The secondary structure is indicated as a cartoon representation. The structures are similar (homologous) but are, in the details, strain-specific (Swiss-model server structures served as a template for the visualization by VMD).

In addition, we applied our own updated protein structure analysis tool AnDOM 2.0 for such complexes (prediction on domains with known structure; see Table S3 in supplementary Material). We used conserved domains with known structure for the protein annotation in structural terms (see Methods and Materials for details). For 13 of the *S. aureus* COL proteins involved in virulence-associated protein complexes (see protein complex overview for wall teichoic acid synthesis in Figure 5) we analyzed their three-dimensional structure, by identifying all domains of these proteins with a known three-dimensional structure. The calculated structures are available as PDB links in Supplementary Material Table S3 with more details on the structure predictions, SCOPE structure information, as well as PDB coordinates and domain pointers to the structures. The virulence-associated protein complexes include enterotoxin complex (enterotoxin G type precursor, SeN, Yent1,2; SeO), cell wall synthesis (glycosyltransferase, wall teichoic acid synthesis), and its regulation (DltB, DltD; [22]). Two further *S. aureus* COL-specific protein structure examples are shown: an ABC transporter (Figure 6) and a lipopolysaccharide core biosynthesis protein (Figure 7). Furthermore, we give a first look on proteins that are strain-specific in clade B (the SecY protein of MRSA252, central part of the SecYEG protein complex) and clade C (Figure 8 and Figure 9).

### 3.3. Detailed Analysis of S. aureus Strain-Specific Proteins

Looking at strain-specific proteins and complexes in more detail, we determined the strain-specific proteins (as predicted from the well-annotated genome sequences) of clade-representative strains against the background of 64 *S. aureus* genomes. Starting from *S. aureus* COL as a central model strain, Figure 2 shows a short distance view considering strain-specific strains for clade A representative strains (COL, HG001, referred to as NCTC8325 due to its complete genome sequence), Newman, and USA300 as well as for clade B (N315). This comparison stresses the conserved proteins and which proteins are, nevertheless, strain-specific even over short phylogenetic distances. Despite their relatively close phylogenetic association, 18 proteins were identified as specific for strain COL, 44 proteins for strain Newman, 67 proteins for strain HG001, 105 proteins for strain N315, and 113 proteins for strain USA300 when taking the core-proteome of all 64 strains into account (data available on request).

Figure 3 shows a “long distance view” considering the resulting strain-specific figures according to a triple comparison between all three clades (A–C), considering COL, N315, and ED133. This triple comparison includes fewer strains, but with higher phylogenetic distance and, hence, stresses differences between individual protein sets of the three strains.

To understand more about the molecular functions encoded by the genome-derived proteome, we considered important protein repertoires involved in virulence, in glycosylation and wall teichoic acid metabolism. Again, this comparison is challenging, here all depending on accurate annotation and proper classification categories. Hence, after rapid comparisons using PERL scripts, all protein comparisons were hand curated. *S. aureus* strains chosen as representatives for the three clades included again for clade A: COL (Genbank accession number: NC_002951), N315 (Genbank accession number: NC_002745), HG001 (Genbank accession number: NC_007795), and USA300_TCH1516 (Genbank accession number: NC_010079). For clade B Newman (Genbank accession number: NC_009641) and Mu50 (Genbank accession number: NC_002758) were chosen, and for clade C we looked at ED133 (Genbank accession number: NC_017337) and MRSA252 (Genbank accession number: NC_002952). Figure 4 gives summary results for the conserved protein complexes found in the strains compared here covering all three clades of *S. aureus* strains. Detailed data are found in Supplementary Table S2. In particular, protein complexes of central metabolism were well-conserved in all of the *S. aureus* strains compared (see discussion). Individual *S. aureus* strains were also directly analyzed for their content of metabolic enzymes. Variation is, again, not too high: the number of annotated metabolic enzymes in *S. aureus* strain COL is 1145, in Mu50 it is 1181, and in ED133 it is 1160. Supplementary Material gives further detailed data, details are found on conserved proteins and protein complexes (Supplementary Excel Table S2) and strain-specific proteins and complexes (Supplementary Tables S5 and S6).

### 3.4. S. aureus COL Proteins

We then focused on strain-specific protein complexes. For this we reanalyzed the calculated list of strain-specific proteins to make predictions of protein complexes. As above, we point out physically interacting protein complexes for all strains, as well as novel predictions based on bioinformatics (which still have to be confirmed by laboratory experiments. Where available, literature references supporting this by experimental data are however given; see Table 2, Figure 5, and following figures). We studied several examples of functional protein complexes and associations in detail, starting with wall teichoic acid metabolism in *S. aureus* COL, as well as other cell wall glycosyltransferases (Figure 5; Table 2). There are 18 strain-specific genes in COL; however, we could easily identify a specific protein complex (SACOL_RS00270, SACOL_RS00275) involved in cell wall structure biosynthesis, which does not occur in the other strains.

Moreover, we studied the individual composition of the *S. aureus*-specific complexes in COL. Supplementary Table S3 shows structural composition and analysis of these complexes using the latest version (v. 2015) of our 3D protein prediction tool AnDOM [10]. For 13 of the protein structures in the COL-specific complexes (Figure 5, Figure 6 and Figure 7) we examined how far a structure prediction is possible comparing PSSMs and HMMs and using a specific database containing all known structural domains (see Methods and Materials for details). For most of the proteins some structure prediction was possible, describing enterotoxins, glycosyltransferases, and a nickel/peptide ABC transporter. As the structures of the ABC transporter (SACOL0694) and the lipopolysaccharide core biosynthesis protein (RfaG, SACOL0052) are potential drug target structures, and could be modeled in detail from identified structure templates, we show them as color figures (Figure 6 and Figure 7, respectively). Details of the structural domain composition are given in Supplemental Table S3. For several proteins where no full homologous protein templates are available, either a SCOP homolog is identified or at least the name of a homologous protein with known 3D structure according to the HMM searches.

### 3.5. S. aureus N315 Proteins

**Figure 10:**
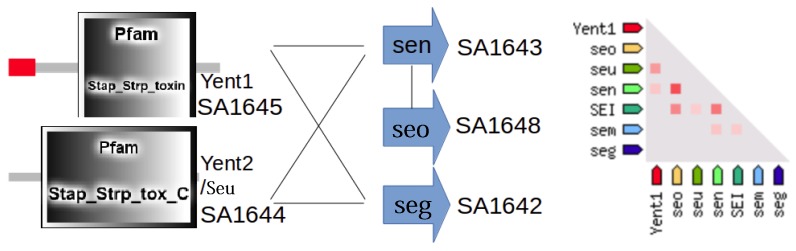
Protein complex of *S. aureus* N315-specific enterotoxins. Strain-specific genes were studied, and the co-occurrence of their orthologs suggests that Yent1 and Yent2 proteins are subunits of a protein complex (left), which further interacts with Sen (SA1643), Seo (SA1648), and Seg (SA1642). The interaction (right) is suggested by circumstantial evidence from the STRING database (see Materials and Methods, Results), for instance co-expression of all components together (except Seg) in many strains analyzed (triangle on the right; intensity of red color indicates strong co-expression). Further evidence from gene context prediction methods points to the interactions shown on the left (Yent1, Yent2/Seu, Sen, Seo, Seg). The red leading region in Yent1 indicates the presence of an intact signal peptide. However, this is no substitute for direct measurements, particularly well-studied is the interaction between Yent1 and Yent2 (see text and final part in Supplementary File S1). The list of strain-specific proteins and sequences is given for this comparison in Supplementary Material File S5.

Enterotoxin genes *yent2* (also known as *seu*, SA1644), *yent1* (SA1645), *seg* (SA1642), *sen* (SA1643), and *seo* (SA1648) could form a complex in *S. aureus* N315 (Figure 10). We predict, according to our bioinformatics prediction by STRING using gene-context, gene fusion, and gene co-occurrence of *yent2* and *yent1*, that their encoded proteins form a complex (evaluated in Supplementary File S1, including other occurrences of *yent1* and *yent2* in *S. aureus* strains). However, as a first observation supporting that the two Yent proteins really form a complex, these two proteins only occur together in *S. aureus* strains, they are SAPI-encoded and if they are absent, they are both absent. Regarding the other proteins Seg (SA1642), Sen (SA1643), and Seo (SA1648), there is some evidence for interaction as suggested by gene neighborhood and homology. Furthermore, the proteins Sen and Seo have also co-expression evidence for interaction. Hence we predict direct physical interaction for proteins Yent1 and Yent2, but only weaker (functional) association for the other three. However, we can probably be even more confident about the complex of the two as Yent1 and Yent2 function only together to yield the functional enterotoxin, otherwise they behave as non-functional pseudogenes [23].

### 3.6. Other S. aureus Strains (Clade B, Clade C)

**Figure 11:**
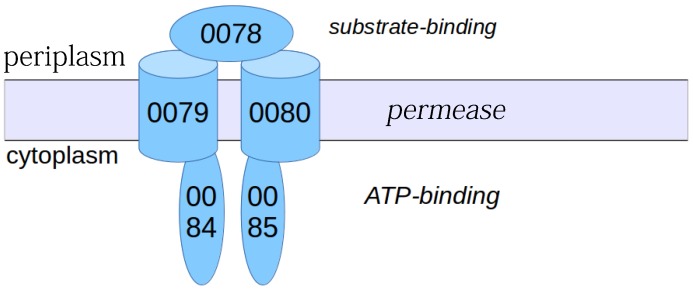
USA300 strain-specific transporter complex. Strain comparisons predict that *S. aureus* USA300_TCH1510 has a specific protein complex, which is actually a nickel peptide ABC transporter consisting of five subunits, encoded by USA300HOU_0078, 0079, 0080, 0084, and 0085. The list of strain-specific proteins and sequences is given in Supplementary File S5.

In other strains the virulence-associated genes form often functional associations. This is sketched for a protein complex in N315 (enterotoxin protein complex, Figure 10; clade B) and, in particular, USA300 (Figure 11; clade A). USA300_TCH1516 [15], a variant from USA300 isolate, has a very interesting strain-specific protein complex which is a nickel/peptide ABC transporter, consisting of five subunits (USA300HOU_0078 to USA300HOU_0082). This may imply a crucial role for USA300 survival; thus, this is a first indication that this may be an important drug target. Another predicted protein complex consists of arginine repressor and universal stress protein (USA300HOU_0071, USA300HOU_0072). The bioinformatics prediction relies here on gene neighborhood and functional considerations (see Materials and Methods). However, phosphoproteome changes give first indications that there is, in fact, a tight functional link between both [21]. Nevertheless, this has, of course, to be complemented by direct biochemical experiments in USA300 to confirm this prediction.

We show detailed structure prediction results for two proteins from strain-specific protein complexes for clade C (Figure 8, MRSA252, SecYEG protein complex) and clade B (Figure 9 Methicillin resistance repressor MecI) applying the AnDOM structure prediction tool. The idea here is to investigate structure predictions for proteins in complexes occurring in clade C and clade B, respectively.

There are several such strain-specific complexes. For instance, N315 strain-specific genes tells us there is a methicillin resistance protein complex (SA_RS00340, SA_RS00345), composed of a beta-lactam sensor and mecA-type methicillin resistance repressor, MecI. That both are transferred together with several other genes involved in methicillin resistance and reside in the bacterial membrane together is necessary for a good response against methicillin [24,25].

Moreover, there is an ABC transporter complex (SA_RS01140, SA_RS01145); the first one is subunit A, the second one, subunit BC. The ABC transporter complex is well-established. Finally, there is a large toxin gene cluster in N315 (SA_RS09240, SA_RS09245, SA_RS09250, SA_RS09255, and SA_RS09270). The genes are direct neighbors, reside in the same location in the chromosome, and are co-expressed. So, at least functionally, they work together; they are also probably found together on the membrane. There is another strain-specific enterotoxin protein in Newman. These enterotoxins are also considered as vaccine targets against *S. aureus* [26].

As a further clade B member, the strain NCTC8325 and its derivative HG001 are endowed with a lot of phage-specific genes and specific transporter units—all these proteins can also be termed parts of their corresponding complex, but these are at present only predictions. To compare clade B-specific protein complexes, we looked at N315 and Mu50 showing strain-specific variation in methicillin resistance repressor MecI protein, a part of the protein complex involved in methicillin resistance (Figure 9).

## 4. Discussion

*S. aureus* is an important model organism and pathogen. Its proteome is well studied [27,28], however, its dynamics and regulation still present challenges and we often lack information on detailed insight into the protein complexes formed. New advances in bioinformatics and systems biology allow us to investigate proteome changes in different dimensions. Starting from comparatively solid ground, we focus on sequence evidence (genome sequences) and model strains and assemble best predictions, biochemical rules, and experimental evidence to show the conserved and strain-specific protein complexes known for eight *S. aureus* strains representing the three clades of *S. aureus* strains. We started from three extensive studies in systems biology to delineate a set of conserved core complexes in *S. aureus* [1,4,8]. As more data come in from more and more detailed proteomics studies, this list will be both extended and refined.

Table 2 illustrates several strain-specific proteins but shows that the overall protein functions and protein complexes for these interesting functions are shared between strains, and even between clades. The backbone of conserved functions (Supplementary File S2) shows that *S. aureus* strains share particularly well their central metabolism, a recurrent theme in bacteria [4]. Enzymes are often well known in their basic structure and, hence, allowed us to model several interesting proteins from virulence-involved protein complexes for *S. aureus* COL. The structure annotation approach used [10] gives the detailed structure as links and pointers to conserved structural domains from which the protein is formed (see materials and methods; detailed results in Supplementary File S3). We annotated every little segment of known structure in these 13 *S. aureus* proteins. However, calculating homology models from such data, the Figure 5, Figure 6, Figure 7, Figure 8, Figure 9, Figure 10 and Figure 11 give more detailed protein structure modelling results on strain-specific proteins from all three *S. aureus* clades as pictures and views on the three dimensional structure of selected *S. aureus* proteins from different protein complexes.

Furthermore, looking at the strictly strain-specific protein lists calculated from extensive sequence comparisons instead (Supplementary Files S5 and S6) shows that the individual strains have proteins involved in membrane functions, mobile genetic elements, and virulence factors, but also a considerable portion of hypothetical proteins still requiring more experimental investigations to understand their specific function.

Analyzing *S. aureus* complexes is challenging as there is also variation in *S. aureus* protein complexes over time and there are different modes to regulate this (Table 2): phosphorylation, glycosylation, and other protein modifications, regulatory interactions include RNA but also accessory proteins, shuttling complexes, metabolites, and the energy state of the cell.

Analysis, calculations of structure, and description of individual complexes are time-consuming and challenging, and it is even more important to complement these observations and predictions with follow-up experiments on the dynamics of protein complexes; a technically challenging undertaking.

Protein complexes change with time and play a crucial role in the adaptation of bacteria which should not be underestimated. Typical situations where this becomes important include adaptation of protein complexes in the diauxic shift [1], and the use of key protein complexes as potential drug targets (Figure 10 and Figure 11). Furthermore, several complexes (e.g., antiporters, ABC transporters, Figure 6 and Figure 11) are heavily involved in adaptation against xenobiotics [13]. Protein modification triggers assembly and modification of protein complexes, for instance, by protein phosphorylation (Table 2), by system adaptation (e.g., aerobic, anaerobic), metabolism, or in ribonucleoproteins (Supplementary File S4), and maybe also by bridging metabolites. Finally, virulence factors are generally expressed condition-specific, for instance enterotoxins (Figure 6, N315 enterotoxin), cell wall synthesis (Figure 5 and Figure 7 for COL), secretion systems (Figure 8, SecY of MRSA252), and methicillin resistance (Figure 9, repressor MecI comparing N315 and Mu50).

One regulatory mechanism involved in the flexibility of protein complexes is post-translational modifications. Modifications, such as protein phosphorylation, glycosylation, and acetylation represent an efficient means to regulate the activity of the individual subunits and, thus, the entire ensemble of proteins as such. As modifying moieties could be rapidly removed or added, protein functionality and/or structure quickly becomes adapted to environmental changes, such as the transition from aerobic to anaerobic conditions. From our data it becomes clear that protein modifications might indeed play a fundamental role in the regulation of protein complexes and their assembly. For the Ser/Thr kinases, for example, we observe that, in contrast to all other strains, the *S. aureus* strain COL expresses a shortened version of the kinase, presumably affecting its modifying activity/specificity. In fact, it has been reported by the Ohlsen group that methicillin resistance is affected if *pknB* (synonym for *stk*, Ser/Thr kinase) is deleted [29].

The phylogenetic analysis points out that the three USA300 strains are assigned to the same clade, but are quite distinct from each other, in particular the first characterized USA300 isolate FPR3757 [30]. The second is TCH1516 [31], which is closer to a recently reported ISMMS1 [32]. This supports the importance of strain variation for these dangerous and highly resistant *S. aureus* variants.

Studying protein complexes in *S. aureus* and their changes is a direct route to identify important switches involved in systems biological adaptation. With these data, more detailed investigations of these *S. aureus* COL protein complexes and protein structures are possible; for instance, detailed investigations on the whole complex, its assembly and disassembly (currently done by us for pyruvate dehydrogenase complex in *S. aureus* COL considering all subunits), or direct targeting of such protein complexes by different drugs, which then requires a systems biological analysis of these drug effects (e.g., [12]).

Regarding clinical relevant isolates such as the USA300 variants examined, our results point out promising targets for direct pharmacological intervention. For instance, to prevent protein complex formation, there is now a range of novel peptide-based or chemically-improved inhibitors available (e.g., [12]). This could be used as new agents against MRSA. In summary, these are further arguments why the study of *S. aureus* protein complexes is both interesting and challenging, and why a general overview on the protein complex repertoire available for *S. aureus* strains is important though it can only focus on selected, but representative examples.

## 5. Conclusions

Protein complexes form a backbone of adaptation. We show the conserved, as well as the strain-specific, protein complexes for eight representative *S. aureus* strains. We look at all three major clades and comparing against a background of 64 strains where full genome information is available. Strain-specific proteins often allow for specific virulence factors and cell wall synthesis. Several such protein structures were examined in detail, annotating domains with known three-dimensional structure and giving selective examples for the full protein repertoire available for *S. aureus* strains established by the extensive all-against-all sequence comparisons. Though we established reliable data combining bioinformatics with data available from literature and databases, much more research is required to completely understand the details of protein complexes and their flexible adaptation in *S. aureus*.

## Figures and Tables

**Table 1:** Basic information on the available data for the strains compared.

Strain Name	Accession	Genome Size	Proteins ^1^	Protein Complexes ^2^
N315	NC_002745	2814816	2714	37
COL	NC_002951	2809422	2764	36
HG001 (NCTC 8325)	NC_007795	2821361	2767	34
USA300_TCH1516	NC_010079	2872915	2660	36
Newman	NC_009641	2878897	2894	35
ED133	NC_017337	2832479	2740	(See results)
Mu50	NC_02758	2878530	2812	
MRSA252	NC_002952	2902620	2781	

^1^ Number of proteins estimated from predicted reading frames; ^2^ Number of complexes estimated according to known complexes and conservation in *S. aureus* COL, taking into account in additional strain-specific protein complexes.

**Table:** **(a)**

Annotation	COL (A)	N315(B)	HG001(A)	Newman(A)	USA300_TCH1516(A)
glycosyltransferase tuaA	SACOL0114	SA0124	SAOUHSC_00089	NWMN_0073	SAUSA300_0131
glycosyltransferase epsF	SACOL0115	SA0125	SAOUHSC_00090	NWMN_0074	SAUSA300_0132
glycosyltransferase fam.1	SACOL0147	SA0155	SAOUHSC_00125	NWMN_0106	SAUSA300_0163
glycosyltransferase tarS	SACOL0243	SA0248	SAOUHSC_00228	NWMN_0192	SAUSA300_0252
glycosyltransferase tagE1	SACOL0611	SA0522	SAOUHSC_00547	NWMN_0526	SAUSA300_0549
glycosyltransferase tagE2	SACOL0612	SA0523	SAOUHSC_00548	NWMN_0527	SAUSA300_0550
Glycosyltransferase tagX	SACOL0697	SA0596	SAOUHSC_00644	NWMN_0610	SAUSA300_0627
glycosyltransferase	SACOL0764	SA0659	SAOUHSC_00713	NWMN_0673	SAUSA300_0689
glycosyltransferase tarM	SACOL1043	-	-	NWMN_0906	SAUSA300_0939
glycosyltransferase	SACOL1498	SA1291	SAOUHSC_01475	NWMN_1369	SAUSA300_1349
glycosyltransferase	SACOL1932	SA1691	SAOUHSC_02012	NWMN_1766	SAUSA300_1855
4,4-diaponeurosporenoate glycosyltransferase	SACOL2578	SA2350	SAOUHSC_02880	NWMN_2463	SAUSA300_2500
accessory Sec system glycosyltransferase GtfB	SACOL2669	SA2440	SAOUHSC_02983	NWMN_2546	SAUSA300_2582
accessory Sec system glycosyltransferase GtfA	SACOL2670	SA2441	SAOUHSC_02984	NWMN_2547	SAUSA300_2583
*N*-glycosyltransferase icaA	SACOL2689	SA2459	SAOUHSC_03002	NWMN_2565	SAUSA300_2600

**Table:** **(b)**

Annotation	COL (A)	Mu50 (B)	ED133 (C)
virulence factor (*esxA*)	SACOL_RS01375	SAV_RS01590	SAOV_RS01175
virulence factor (*esxB*)	SACOL_RS01410	SAV_RS01625	SAOV_RS01210
virulence factor B (*cvfB*)	SACOL_RS07265	SAV_RS07495	SAOV_RS07490
virulence factor C	SACOL_RS07475	SAV_RS07700	
putative enterotoxin	SACOL_RS02230	SAV_RS02045	SAOV_RS02015
leucotoxin LukDv (*lukD*)	SACOL_RS09650	SAV_RS09765	SAOV_RS09495
Enterotoxin1 (*sek*)	SACOL_RS04550	SAV_RS09795	SAOV_RS02205
enterotoxin (*sei*)	SACOL_RS04555	SAV_RS09800	
enterotoxin (*seb*)	SACOL_RS04655	SAV_RS09805	SAOV_RS02280
enterotoxin			SAOV_RS05800
enterotoxin type A	SACOL_RS08455	SAV_RS09810	SAOV_RS08355
enterotoxin (*epiD*)	SACOL_RS09620	SAV_RS09815	SAOV_RS09460
enterotoxin (*sem*)		SAV_RS09820	
enterotoxin (*seo*)		SAV_RS09825	
enterotoxin (*sep*)		SAV_RS10675	
enterotoxin (*sel*)		SAV_RS10975	SAOV_RS02210
enterotoxin (*sec3*)		SAV_RS10980	
antitoxin MazE (*mazE*)	SACOL_RS10780	SAV_RS11325	SAOV_RS11100
toxin	SACOL_RS11560	SAV_RS12060	SAOV_RS11860
Antitoxin RelB (*relB*)	SACOL_RS12615	SAV_RS13100	SAOV_RS12895
MarF (*marF*)	SACOL_RS01610		

**Table:** (**c**)

Gene	Annotation	N315 (B)	COL (A)	HG001 (A)	Newman (A)	USA300TCH1516 (B)
*stk*	serine/threonine kinase	SA1063	Partial (388/664)	SAOUHSC_01187	NWMN_1130	SAUSA300_1113
*stp*	serine/threonine phosphatase	SA1062	SACOL1231	SAOUHSC_01186	NWMN_1129	SAUSA300_1112
	2, 3-cyclic nucleotide 2-phosphodiesterase	SA0140	SACOL0130	SAOUHSC_00107	NWMN_0088	SAUSA300_0147
	DNA repair exonuclease	SA1662	SACOL1900	SAOUHSC_01975	NWMN_1736	SAUSA300_1793
	phosphohydrolase	SA2225	SACOL2440	SAOUHSC_02728	NWMN_2336	SAUSA300_2382

## Supplementary Materials

The following are available online at www.mdpi.com/2227-7382/4/1/8/s1, Supplementary File S1 Overview to supplementary data with Figure S1, Completely sequenced *S. aureus* genomes and clades used in this study; Supplementary File and Table S2, Protein complexes conserved in *S. aureus*; Table S3, Top structure results generated with AnDOM and HH-suite for *S. aureus*; Table S4, Examples for how Protein complexes may be modified (time, post-translational, ribonucleoproteins); Table S5, Strain-specific proteins of five model strains; Table S6, Strain-specific proteins of three model strains for three different clades.

## References

[1] Liang C., Liebeke M., Schwarz R., Zühlke D., Fuchs S., Menschner L., Engelmann S., Wolz C., Jaglitz S., Bernhardt J. (2011). *Staphylococcus aureus* physiological growth limitations: Insights from flux calculations built on proteomics and external metabolite data. Proteomics.

[2] Keseler I.M., Mackie A., Peralta-Gil M., Santos-Zavaleta A., Gama-Castro S., Bonavides-Martínez C., Fulcher C., Huerta A.M., Kothari A., Krummenacker M. (2013). EcoCyc: Fusing model organism databases with systems biology. Nucleic Acids Res..

[3] Cafarelli T.M., Rands T.J., Godoy V.G. (2014). The DinB•RecA complex of *Escherichia coli* mediates an efficient and high-fidelity response to ubiquitous alkylation lesions. Environ. Mol. Mutagen..

[4] Krüger B., Liang C., Prell F., Fieselmann A., Moya A., Schuster S., Völker U., Dandekar T. (2012). Metabolic adaptation and protein complexes in prokaryotes. Metabolites.

[5] Hecker M., Becher D., Fuchs S., Engelmann S. (2010). A proteomic view of cell physiology and virulence of *Staphylococcus aureus*. Int. J. Med. Microbiol..

[6] Hecker M., Reder A., Fuchs S., Pagels M., Engelmann S. (2009). Physiological proteomics and stress/starvation responses in *Bacillus subtilis* and *Staphylococcus aureus*. Res. Microbiol..

[7] Altschul S.F., Gish W., Miller W., Myers E.W., Lipman D.J. (1990). Basic local alignment search tool. J. Mol. Biol..

[8] Kühner S., van Noort V., Betts M.J., Leo-Macias A., Batisse C., Rode M., Yamada T., Maier T., Bader S., Beltran-Alvarez P. (2009). Proteome organization in a genome-reduced bacterium. Science.

[9] Szklarczyk D., Franceschini A., Wyder S., Forslund K., Heller D., Huerta-Cepas J., Simonovic M., Roth A., Santos A., Tsafou K.P. (2015). STRING v10: Protein-protein interaction networks, integrated over the tree of life. Nucleic Acids Res..

[10] Schmidt S., Bork P., Dandekar T. (2002). A versatile structural domain analysis server using profile weight matrices. J. Chem. Inf. Comput. Sci..

[11] Liang C., Krüger B., Dandekar T. (2013). GoSynthetic database tool to analyse natural and engineered molecular processes. Database (Oxford).

[12] Cecil A., Ohlsen K., Menzel T., François P., Schrenzel J., Fischer A., Dörries K., Selle M., Lalk M., Hantzschmann J. (2015). Modelling antibiotic and cytotoxic isoquinoline effects in *Staphylococcus aureus*, *Staphylococcus epidermidis* and mammalian cells. Int. J. Med. Microbiol..

[13] Cecil A., Rikanović C., Ohlsen K., Liang C., Bernhardt J., Oelschlaeger T.A., Gulder T., Bringmann G., Holzgrabe U., Unger M. (2011). Modeling antibiotic and cytotoxic effects of the dimeric isoquinoline IQ-143 on metabolism and its regulation in *Staphylococcus aureus*, *Staphylococcus epidermidis* and human cells. Genome Biol..

[14] Andreeva A., Howorth D., Brenner S.E., Hubbard T.J.P., Chothia C., Murzin A.G. (2004). SCOP database in 2004: Refinements integrate structure and sequence family data. Nucleic Acids Res..

[15] Schäffer A.A., Wolf Y.I., Ponting C.P., Koonin E.V., Aravind L., Altschul S.F. (1999). IMPALA: Matching a protein sequence against a collection of PSI-BLAST-constructed position-specific score matrices. Bioinformatics.

[16] FTP site of National Center for Biotechnology Information. ftp://ftp.ncbi.nih.gov.

[17] Smith T.F., Waterman M.S. (1981). Identification of Common Molecular Subsequences. J. Mol. Biol..

[18] Guindon S., Dufayard J.F., Lefort V., Anisimova M., Hordijk W., Gascuel O. (2010). New algorithms and methods to estimate maximum-likelihood phylogenies: assessing the performance of PhyML 3.0. Syst. Biol..

[19] Tong S.Y., Schaumburg F., Ellington M.J., Corander J., Pichon B., Leendertz F., Bentley S.D., Parkhill J., Holt D.C., Peters G. (2015). Novel staphylococcal species that form part of a *Staphylococcus aureus*-related complex: The non-pigmented *Staphylococcus argenteus* sp. nov. and the non-human primate-associated *Staphylococcus schweitzeri* sp. nov.. Int. J. Syst. Evol. Microbiol..

[20] Baba T., Bae T., Schneewind O., Takeuchi F., Hiramatsu K. (2008). Genome sequence of *Staphylococcus aureus* strain Newman and comparative analysis of staphylococcal genomes: Polymorphism and evolution of two major pathogenicity islands. J. Bacteriol..

[21] Bäsell K., Otto A., Junker S., Zühlke D., Rappen G.M., Schmidt S., Hentschker C., Macek B., Ohlsen K., Hecker M. (2014). The phosphoproteome and its physiological dynamics in *Staphylococcus aureus*. Int. J. Med. Microbiol..

[22] Koprivnjak T., Mlakar V., Swanson L., Fournier B., Peschel A., Weiss J.P. (2006). Cation-Induced Transcriptional Regulation of the dlt Operon of *Staphylococcus aureus*. J. Bacteriol..

[23] Heymans F., Fischer A., Stow N.W., Girard M., Vourexakis Z., Des Courtis A., Renzi G., Huggler E., Vlaminck S., Bonfils P. (2010). Screening for staphylococcal superantigen genes shows no correlation with the presence or the severity of chronic rhinosinusitis and nasal polyposis. PLoS ONE.

[24] Paterson G.K., Harrison E.M., Holmes M.A. (2014). The emergence of *mecC* methicillin-resistant *Staphylococcus aureus*. Trends Microbiol..

[25] Harrison E.M., Paterson G.K., Holden M.T., Morgan F.J., Larsen A.R., Petersen A., Leroy S., de Vliegher S., Perreten V., Fox L.K. (2013). A *Staphylococcus xylosus* isolate with a new mecC allotype. Antimicrob. Agents Chemother..

[26] Pinchuk I.V., Beswick E.J., Reyes V.E. (2010). Staphylococcal enterotoxins. Toxins (Basel).

[27] Kohler C., Wolff S., Albrecht D., Fuchs S., Becher D., Büttner K., Engelmann S., Hecker M. (2005). Proteome analyses of Staphylococcus aureus in growing and non-growing cells: A physiological approach. Int. J. Med. Microbiol..

[28] Fuchs S., Zühlke D., Pané-Farré J., Kusch H., Wolf C., Reiß S., le Binh T.N., Albrecht D., Riedel K., Hecker M. (2013). Aureolib—A proteome signature library: Towards an understanding of *staphylococcus aureus* pathophysiology. PLoS ONE.

[29] Ohlsen K., Donat S. (2010). The impact of serine/threonine phosphorylation in *Staphylococcus*
*aureus*. Int. J. Med. Microbiol..

[30] Diep B.A., Gill S.R., Chang R.F., Phan T.H., Chen J.H., Davidson M.G., Lin F., Lin J., Carleton H.A., Mongodin E.F. (2006). Complete genome sequence of USA300, an epidemic clone of community-acquired meticillin-resistant *Staphylococcus aureus*. Lancet.

[31] Highlander S.K., Hultén K.G., Qin X., Jiang H., Yerrapragada S., Mason E.O., Shang Y., Williams T.M., Fortunov R.M., Liu Y. (2007). Subtle genetic changes enhance virulence of methicillin resistant and sensitive *Staphylococcus aureus*. BMC Microbiol..

[32] Altman D.R., Sebra R., Hand J., Attie O., Deikus G., Carpini K.W., Patel G., Rana M., Arvelakis A., Grewal P. (2014). Transmission of methicillin-resistant *Staphylococcus aureus* via deceased donor liver transplantation confirmed by whole genome sequencing. Am. J. Transplant..

