# Supplementary material for: A Staphylococcus aureus Proteome Overview: Shared and Specific Proteins and Protein Complexes from Representative Strains of All Three Clades
Source: Proteomes. 2016 Feb 19;4(1):8. doi: 10.3390/proteomes4010008 (PMC5217359; doi:10.3390/proteomes4010008)
Supplement: Supplementary file 1 [file proteomes-04-00008-s001.zip › proteomes-04-00008-supplementary/Supplementary File S1-general-and-strains.pdf]

proteomes

Supplementary File S1

A short guide to all Supplementary data files

This guide helps to make the study more accessible and reproducible for the interested reader by providing further detailed data on the results shown in the main paper. It has direct pointers to the genome and proteome comparison data so that all strains can be compared with each other:

We include next in this file a list of all *S. aureus* genomes and their characteristics. After this, their clade distribution follows (Supplementary Table S1). Finally, we reexamine the STRING prediction for *yent1* and 2 for illustration.

In order to keep the study accessible and reproducible, we add for all the conserved protein complexes, Supplementary Table S2, to the supplement. It shows all the proteins (rows) and the strain-specific information in which they occur (columns). As creation of this Table is quite time-consuming (first, do an all-against-all protein comparison among all strains, then identify and annotate conserved proteins, then identify and annotate conserved protein complexes). This table of conserved protein complexes had to be restricted to the first five strains compared. However, the genome comparisons we did so far suggest that these protein complexes are, in fact, found in most of the *S.aureus* strains we know.

However, we give next detailed information on all strain-specific proteins:

We first studied the individual composition of the *S. aureus*-specific complexes in COL. Supplementary Table S3 shows structural composition and analysis of these complexes using the latest version (v. 2015) of our 3D protein prediction tool AnDOM. For 13 of the protein structures in the COL-specific complexes (Figures 5, 8, and 9) we examined how far a structure prediction is possible comparing PSSMs and HMMs and, using a specific database containing all known structural domains, (see Materials and Methods for details). The table contains all information on conserved structure domains found in the infection-relevant *S. aureus* proteins analyzed by AnDOM. For most of the proteins some structure prediction was possible, describing enterotoxins, glycosyltransferases, and a nickel/peptide ABC transporter. The AnDOM predictions in this Supplementary File S4 include the alignment and direct pointers to the connected known 3D structures. Furthermore, predictions from the HH-suite software are given in the table. The most-recent database of non-redundant proteins provided (90 percent sequence identity; date of creation: August of 2011 [1]) was used for sequence augmentation of *S. aureus* sequences in FASTA format. The HH-suite methodology is based on an HMM-HMM comparison algorithm which offers highest sensitivity in the detection of distant homologs and creates alignments with high accuracy [2]. The parameters were defined to create sequence alignments with highest-possible diversity out of of the available sequence database. HHblits performed five search iterations with an elevated level of the “neffmax” parameter, which controls target diversity of the resulting alignments.

Next we point out different biological mechanisms leading to dynamic changes in bacterial protein complexes by system adaptation (e.g. aerobic, anaerobic), metabolism, or in ribonucleoproteins (Supplementary File S4).

We compared five strains, COL, HG001, Newman, and USA300 (clade A) to N315 (clade B) regarding their strain-specific proteome (Figure 2). Supplementary File S5 is an Excel file and Table which shows all strain-specific proteins found for each strain regarding this comparison.

Figure 3 shows the proteome comparison between three representative *S. aureus* strains from the three major clades against the background of all strains (*S. aureus* COL (clade A), Mu50 (clade B), and ED133 (clade C). Again, the information for each strain and all its specific proteins is given: Supplementary File S6 is an Excel file and Table, which shows all strain-specific proteins found for this second comparison; several strain-specific complexes are highlighted.

Table S1. Completely sequenced *S. aureus* genomes used in this study.

Clade	*S. aureus* Strains	Accession
A	USA300-ISMMS1	NZ_CP007176
A	VC40	NC_016912
A	ATCC_BAA1680_29b_MRSA	NC_009641
A	Newman	NC_009641
A	NRS100	NZ_CP007539
A	COL	NC_002951
A	ATCC_BAA1680_33b_MRSA	NZ_CP010297
A	USA300_FPR3757	NC_007793
A	2395_USA500	NZ_CP007499
A	ATCC_BAA1680_27b_MRSA	NZ_CP010300
A	DSM_20231	NZ_CP011526
A	CA15	NZ_CP007674
A	ATCC_BAA1680_31b_MRSA	NZ_CP010296
A	Z172	NC_022604
A	T0131	NC_017347
A	Gv69	NZ_CP009681
A	TW20	NC_017331
A	Bmb9393	NC_021670
A	M121	NZ_CP007670
A	USA300_TCH1516	NC_010079
A	ATCC_BAA1680_26b_MRSA	NZ_CP010298
A	NCTC_8325	NC_007795
A	ATCC_BAA1680_25b_MRSA	NZ_CP010299
B	502A_RN6607	NZ_CP007454
B	ECT-R_2	NC_017343
B	Mu50	NC_002758
B	Mu3	NC_009782
B	ST228_16035	NC_020533
B	ST228_18412	NC_020537
B	ST228_10388	NC_020529
B	ST228_18583	NC_020568
B	ST228_10497	NC_020532
B	ST228_18341	NC_020536
B	ST228_16125	NC_020566
B	ED98	NC_013450
B	04-02981	NC_017340
B	N315	NC_002745
B	FCFHV36	NZ_CP011147
B	JH1	NC_009632
B	JH9	NC_009487
B	CN1	NC_022226
B	11819-97	NC_017351
B	H-EMRSA-15	NZ_CP007659
B	HO_5096_0412	NC_017763
B	RKI4	NZ_CP011528
B	ST772-MRSA-V_DAR4145	NZ_CP010526
B	MSSA476	NC_002953
B	MW2	NC_003923
C	08BA02176	NC_018608
C	ST398_S0385	NC_017333

C	6850	NC_022222
C	SA268	NZ_CP006630
C	SA957	NC_022442
C	M013	NC_016928
C	SA40	NC_022443
C	JKD6159	NC_017338
C	RF122	NC_007622
C	CA-347	NC_021554
C	FORC_001	NZ_CP009554
C	TCH60_MRSA_TCH60	NC_017342
C	ATCC_25923	NZ_CP009361
C	MRSA252	NC_002952
C	ED133	NC_017337
C	LGA251	NC_017349

Reexamining STRING Predictions

Enterotoxin genes *yent2* (*seu*, SA1644), *yent1* (SA1645), *seg* (SA1642), *sen* (SA1643), and *seo* (SA1648) are shown in Figure 6 of the paper. Here we reevaluate the STRING predictions relying on gene-context, gene fusion, and gene co-occurrence that *yent2* and *yent1* form a complex. First of all, these two proteins occur in several *S. aureus* strains, but in many they do not. In the STRING database there are only two strains reported, Mu50 and MSHR1132 (clade B, see below).

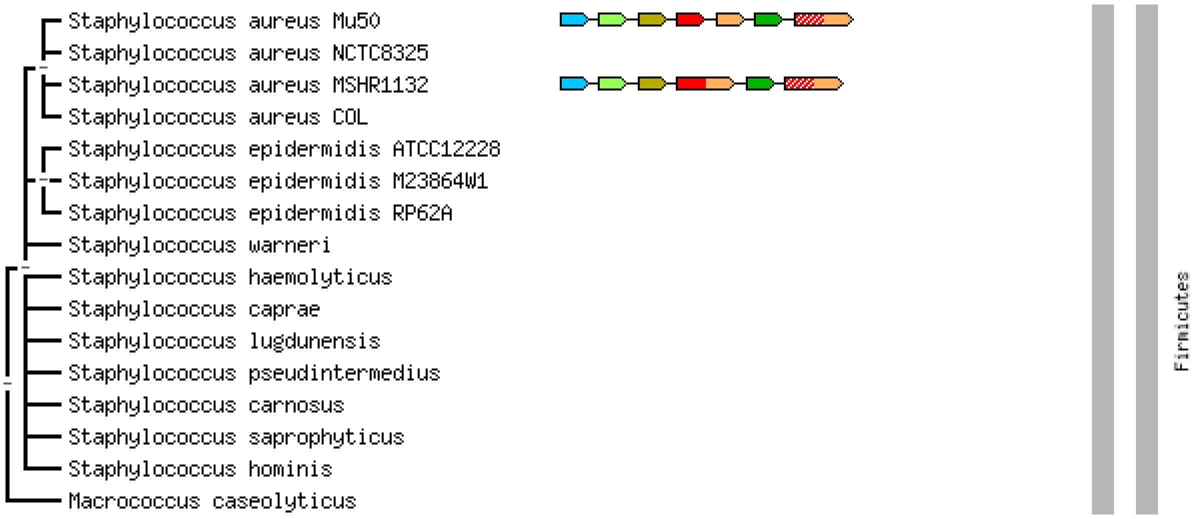

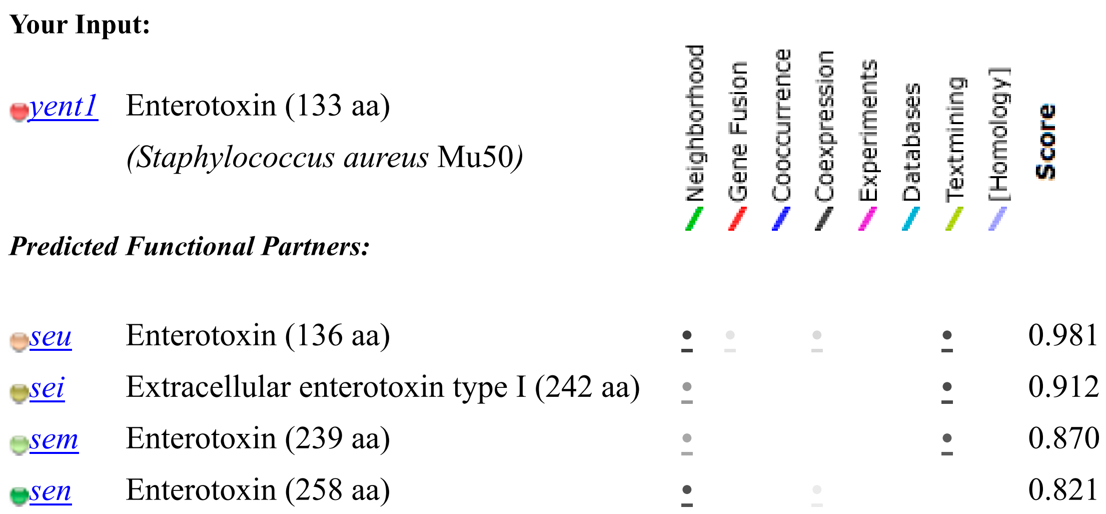

set15	Superantigen-like protein (227 aa)	0.756
sel	Extracellular enterotoxin L (240 aa)	0.753
seo	Enterotoxin (260 aa)	0.685
set10	Superantigen-like protein 5 (234 aa)	0.578
lpl8	Hypothetical protein (260 aa)	0.577
set13	Superantigen-like protein (232 aa)	0.577

Furthermore, though neighborhood supports several more proteins to be in the enterotoxin complex, only for *seu* (*yent2*) there is further evidence from gene fusion and co-occurrence. However, the STRING database compares the evidence it has for a strain-specific complex against a background of more than 2000 organisms (the database currently covers 9,643,763 proteins from 2031 organisms in the current version (Version 10, start of 2016)). Thus, the prediction of an interaction of yent1 and yent2 has nevertheless a high confidence level (0.981). The most right column indicates that, in particular, searching the literature can add some more solid evidence. Hence, we then gave further supporting information from literature in the results: as a first observation supporting that the two yent proteins really form a complex, these two proteins only occur together in *S. aureus* strains, they are SAPI-encoded and, if they are absent, they are both absent. Regarding the other proteins Seg (SA1642), Sen (SA1643), and Seo (SA1648), there is some evidence for interaction as suggested by gene neighborhood and homology. Furthermore, the proteins Sen and Seo have also co-expression evidence for interaction. Hence, we predict direct physical interaction for proteins Yent1 and Yent2, but only weaker (functional) association for the other three. However, we can probably be even more confident about the complex of the two as Yent1 and Yent2 only function together to yield the functional enterotoxin, otherwise they behave as non-functional pseudogenes (see Ref [3,4])

Reference

1. Remmert, M.; Biegert, A.; Hauser, A.; Söding, J. HHblits: lightning-fast iterative protein sequence searching by HMM-HMM alignment. *Nat. Methods* 2011, 9, 173–175.
2. Söding, J. Protein homology detection by HMM-HMM comparison. *Bioinformatics* 2005, 21(7), 951–960.
3. Heymans, F.; Fischer, A.; Stow, N.W.; Girard, M.; Vourexakis, Z.; Des Courtis, A.; Renzi, G.; Huggler, E.; Vlaminck, S.; Bonfils, P.; *et al.* Screening for staphylococcal superantigen genes shows no correlation with the presence or the severity of chronic rhinosinusitis and nasal polyposis. *PLoS ONE* **2010**, *5*, e9525.
4. Letertre, C.; Perelle, S.; Dilasser, F.; Fach, P. Identification of a new putative enterotoxin Seu encoded by the *egc* cluster of *Staphylococcus aureus*. *J Appl Microbiol* **2003**, 95, 38–43.

© 2016 by the authors; licensee MDPI, Basel, Switzerland. This article is an open access article distributed under the terms and conditions of the Creative Commons by Attribution (CC-BY) license (http://creativecommons.org/licenses/by/4.0/).
