# Supplementary material for: A Staphylococcus aureus Proteome Overview: Shared and Specific Proteins and Protein Complexes from Representative Strains of All Three Clades
Source: Proteomes. 2016 Feb 19;4(1):8. doi: 10.3390/proteomes4010008 (PMC5217359; doi:10.3390/proteomes4010008)
Supplement: Supplementary file 1 [file proteomes-04-00008-s001.zip › proteomes-04-00008-supplementary/Supplementary File S3-Table S3-S-aureusCOL-complex-structures.pdf]

Table S3: Top results generated with AnDom webserver and HH-suite for chosen candidates of S. aureus.
SACOL0051, SACOL0052, SACOL0238, SACOL0239 and SACOL0694 show distant homology to known proteins with regulatory domains.

S. aureus candidates			AnDom 2.0 webserver				HH-suite				Additional information
**Identifier**	**Description**	**Sequence**	**1. result**	**2. result**	**3. result**	**4. result**	**1. result**	**2. result**	**3. result**	**4. result**	**Regulatory domains**
**SA1642**	**Extracellular enterotoxin type G precursor**	MKKLSTVIIILILEIVFHNMNYVNAQPDPKLDELNKVSDYKNNKGTMGNVMNLYTSPPVE<br>GRGVINSRQFLSHDLIFPIEYKSYNEVKTELENTELANNYKDKKVDIFGVPYFYTCIIPK<br>SEPDINQNFGGCCMYGGLTFNSSENERDKLITVQVTIDNRQSLGFTITTNKNMVTIQELD<br>YKARHWLTKEKKLYEFDGSAFESGYIKFTEKNNTSFWFDLFPKKELVPFVPYKFLNIYGD<br>NKVVDSKSIKMEVFLNTH	**SCOP: b.40.2.2**<br>Query: 25 - 139 / 258<br>Template: 2 - 119 / 121<br>E-value = 1e-26<br><br>**Staphylococcal enterotoxin B, SEB {Staphylococcus aureus [TaxId: 1280]}**	**SCOP: d.15.6.1**<br>Query: 146 - 256 / 258<br>Template: 2 - 109 / 109<br>E-value = 1e-24<br><br>**Streptococcal superantigen Spe-H {Streptococcus pyogenes [TaxId: 1314]}**	**SCOP: b.40.2.2**<br>Query: 141 - 257 / 258<br>Template: 1 - 115 / 117<br>E-value = 2e-23<br><br>**Staphylococcal enterotoxin B, SEB {Staphylococcus aureus [TaxId: 1280]}**	**SCOP: b.40.2.2**<br>Query: 147 - 257 / 258<br>Template: 1 - 112 / 113<br>E-value = 6e-22<br><br>**Staphylococcal enterotoxin A, SEA {Staphylococcus aureus [TaxId: 1280]}**	Query: 1 - 255 / 258<br>Template: 10 - 247 / 261<br>E-value = 1.9e-75<br><br>**SEH; Scarlet fever toxin; SPE A; Erythrogenic toxin; Exotoxin type A; SEA.**	Query: 47 - 256 / 258<br>Template: 30 - 237 / 247<br>E-value = 1.3e-51<br><br>**Chain B, Crystal Structure Of Sek; Chain A, Crystal Structure Of Sek; Chain A, Crystal Structure Of Sek-Hvb5.1…**	Query: 87 - 256 / 258<br>Template: 1 - 165 / 167<br>E-value = 2e-50<br><br>**Enterotoxin A; enterotoxin-like toxin type P; enterotoxin D; enterotoxin E; Enterotoxin; enterotoxin B.**	Query: 24 - 235 / 258<br>Template: 21 - 220 / 220<br>E-value = 9e-50<br><br>**Type A exotoxin. [Streptococcus pyogenes]l 47328.**	No homologous regulatory domains found
**SA1643**	**Enterotoxin SeN**	MKNIKKLMRLFYIAAIIITLLCLINNNYVNAEVDKKDLKKKSDLDSSKLFNLTSYYTDIT<br>WQLDESNKISTDQLLNNTIILKNIDISVLKTSSLKVEFNSSDLANQFKGKNIDIYGLYFG<br>NKCVGLTEEKTSCLYGGVTIHDGNQLDEEKVIGVNVFKDGVQQEGFVIKTKKAKVTVQEL<br>DTKVRFKLENLYKIYNKDTGNIQKGCIFFHSHNHQDQSFYYDLYNVKGSVGAEFFQFYSD<br>NRTVSSSNYHIDVFLYKD	**SCOP: d.15.6.1**<br>Query: 147 - 255 / 258<br>Template: 2 - 108 / 109<br>E-value = 6e-24<br><br>**Streptococcal superantigen Spe-H {Streptococcus pyogenes [TaxId: 1314]}**	**SCOP: d.15.6.1**<br>Query: 142 - 257 / 258<br>Template: 1 - 115 / 117<br>E-value = 8e-23<br><br>**Staphylococcal enterotoxin B, SEB {Staphylococcus aureus [TaxId: 1280]}**	**SCOP: d.15.6.1**<br>Query: 148 - 257 / 258<br>Template: 1 - 112 / 113<br>E-value = 5e-22<br><br>**Staphylococcal enterotoxin A, SEA {Staphylococcus aureus [TaxId: 1280]}**	**SCOP: b.40.2.2**<br>Query: 50 - 146 / 258<br>Template: 24 - 119 / 120<br>E-value = 5e-22<br><br>**Staphylococcal enterotoxin A, SEA {Staphylococcus aureus [TaxId: 1280]}**	Query: 7 - 258 / 258<br>Template: 9 - 252 / 261<br>E-value = 2.5e-74<br><br>**SEH; Scarlet fever toxin; SPE A; Erythrogenic toxin; Exotoxin type A; SEA.**	Query: 7 - 255 / 258<br>Template: 1 - 236 / 247<br>E-value = 1.1e-56<br><br>**Chain B, Crystal Structure Of Sek; Chain A, Crystal Structure Of Sek; Chain A, Crystal Structure Of Sek-Hvb5.1…**	Query: 94 - 258 / 258<br>Template: 1 - 167 / 167<br>E-value = 7.9e-53<br><br>**Enterotoxin A; enterotoxin-like toxin type P; enterotoxin D; enterotoxin E; Enterotoxin; enterotoxin B.**	Query: 14 - 257 / 258<br>Template: 15 - 262 / 262<br>E-value = 9.2e-53<br><br>**Enterotoxin O; staphylococcal/Streptococcal toxin, beta-grasp domain protein; toxin OB-fold domain protein; toxin, OB-fold domain protein…**	No homologous regulatory domains found
**SA1644**	**Enterotoxin YENT2**	MKKTCMYGGVTEHDGNQIDKNNSTDNSHNILIKVYENERNSLSFDIPTNKKNITAQEIDY<br>KVRNYLLKHKNLYEFNSSPYETGYIKFIEGSGHSFWYDLMPESGKKFYPTKYLLIYNDNK<br>TVESKSINVEVHLTKK	**SCOP: d.15.6.1**<br>Query: 21 - 136 / 136<br>Template: 2 - 116 / 117<br>E-value = 2e-25<br><br>**Staphylococcal enterotoxin B, SEB {Staphylococcus aureus [TaxId: 1280]}**	**SCOP: d.15.6.1**<br>Query: 25 - 134 / 136<br>Template: 2 - 109 / 109<br>E-value = 8e-23<br><br>**Streptococcal superantigen Spe-H {Streptococcus pyogenes [TaxId: 1314]}**	**SCOP: d.15.6.1**<br>Query: 27 - 135 / 136<br>Template: 2 - 112 / 113<br>E-value = 5e-22<br><br>**Staphylococcal enterotoxin A, SEA {Staphylococcus aureus [TaxId: 1280]}**	**SCOP: d.15.6.1**<br>Query: 25 - 134 / 136<br>Template: 4 - 111 / 112<br>E-value = 5e-22<br><br>**Staphylococcal enterotoxin H, SEH {Staphylococcus aureus [TaxId: 1280]}**	Query: 2 - 134 / 136<br>Template: 125-248 / 261<br>E-value = 4.6e-46<br><br>**SEH; Scarlet fever toxin; SPE A; Erythrogenic toxin; Exotoxin type A; SEA.**	Query: 2 - 136 / 136<br>Template: 37-167 / 167<br>E-value = 9.1e-45<br><br>**Enterotoxin A; enterotoxin-like toxin type P; enterotoxin D; enterotoxin E; Enterotoxin; enterotoxin B.**	Query: 3 - 134 / 136<br>Template: 99-237 / 247<br>E-value = 6.7e-40<br><br>**Chain B, Crystal Structure Of Sek; Chain A, Crystal Structure Of Sek; Chain A, Crystal Structure Of Sek-Hvb5.1…**	Query: 1 - 136 / 136<br>Template: 1-136 / 136<br>E-value = 1e-39<br><br>**YENT2; enterotoxin type C-2; staphylococcal enterotoxin Yent1; enterotoxin type C3; staphylococcal enterotoxin C3; enterotoxin YENT2.**	No homologous regulatory domains found
**SA1645**	**Enterotoxin Yent1**	MKLFAFIFICVKSCSLLFMLNGNPKPEQLNKASEFTGLMDNMRYLYDDKHVSETNIKSQE<br>KFLQHDLLFKINGSKILKTEFNNKSLSDKYKNKNVDLFGTNYYNQCYFSLDNMELNDGRL<br>IEKNVYVWRCDRA	**SCOP: b.40.2.2**<br>Query: 23 - 125 / 133<br>Template: 5 - 112 / 121<br>E-value = 4e-26<br><br>**Staphylococcal enterotoxin B, SEB {Staphylococcus aureus [TaxId: 1280]}**	**SCOP: b.40.2.2**<br>Query: 29 - 113 / 133<br>Template: 2 - 88 / 100<br>E-value = 5e-17<br><br>**Staphylococcal enterotoxin H, SEH {Staphylococcus aureus [TaxId: 1280]}**	**SCOP: b.40.2.2**<br>Query: 21 - 106 / 133<br>Template: 4 - 96 / 120<br>E-value = 8e-17<br><br>**Staphylococcal enterotoxin A, SEA {Staphylococcus aureus [TaxId: 1280]}**	**SCOP: b.40.2.2**<br>Query: 39 - 106 / 133<br>Template: 3 - 70 / 83<br>E-value = 5e-15<br><br>**Enterotoxin type I, SEI {Staphylococcus aureus [TaxId: 1280]}**	Query: 1 - 112 / 133<br>Template: 10 - 123 / 261<br>E-value = 1.1e-42<br><br>**SEH; Scarlet fever toxin; SPE A; Erythrogenic toxin; Exotoxin type A; SEA.**	Query: 1 - 133 / 133<br>Template: 10 - 142 / 156<br>E-value = 8.4e-40<br><br>**YENT1; enterotoxin C3; enterotoxin Yent1; enterotoxin; LOW QUALITY PROTEIN: enterotoxin; enterotoxin Seu.**	Query: 19 - 133 / 133<br>Template: 1 - 115 / 115<br>E-value = 7.3e-38<br><br>**LOW QUALITY PROTEIN: enterotoxin; staphylococcal/ Streptococcal toxin, OB-fold domain protein; staphylococcal enterotoxin Yent2.**	Query: 6 - 113 / 133<br>Template: 17 - 129 / 214<br>E-value = 2.4e-36<br><br>**Enterotoxin C2; enterotoxin SEG variant; enterotoxin; enterotoxin G.**	No homologous regulatory domains found
**SA1648**	**Enterotoxin SeO**	MKNSKVMLNVLLLILNLIAICSVNNAYANEEDPKIESLCKKSSVDPIALHNINDDYINNR<br>FTTVKSIVSTTEKFLDFDLLFKSINWLDGISAEFKDLKVEFSSSAISKEFLGKTVDIYGV<br>YYKAHCHGEHQVDTACTYGGVTPHENNKLSEPKNIGVAVYKDNVNVNTFIVTTDKKKVTA<br>QELDIKVRTKLNNAYKLYDRMTSDVQKGYIKFHSHSEHKESFYYDLFYIKGNLPDQYLQI<br>YNDNKTIDSSDYHIDVYLFT	**SCOP: d.15.6.1**<br>Query: 145 - 260 / 260<br>Template: 1 - 115 / 117<br>E-value = 8e-26<br><br>**Staphylococcal enterotoxin B, SEB {Staphylococcus aureus [TaxId: 1280]}**	**SCOP: d.15.6.1**<br>Query: 151 - 258 / 260<br>Template: 3 - 108 / 109<br>E-value = 8e-26<br><br>**Streptococcal superantigen Spe-H {Streptococcus pyogenes [TaxId: 1314]}**	**SCOP: b.40.2.2**<br>Query: 29 - 150 / 260<br>Template: 3 - 120 / 120<br>E-value = 3e-25<br><br>**Staphylococcal enterotoxin A, SEA {Staphylococcus aureus [TaxId: 1280]}**	**SCOP: d.15.6.1**<br>Query: 151 - 260 / 260<br>Template: 1 - 112 / 113<br>E-value = 2e-24<br><br>**Staphylococcal enterotoxin A, SEA {Staphylococcus aureus [TaxId: 1280]}**	Query: 25 - 260 / 260<br>Template: 25 - 251 / 261<br>E-value = 1.2e-72<br><br>**SEH; Scarlet fever toxin; SPE A; Erythrogenic toxin; Exotoxin type A; SEA.**	Query: 1 - 260 / 260<br>Template: 3 - 262 / 262<br>E-value = 7.2e-63<br><br>**Enterotoxin O; staphylococcal/ Streptococcal toxin, beta-grasp domain protein; toxin OB-fold domain protein…**	Query: 97 - 260 / 260<br>Template: 1-166 / 167<br>E-value = 1.4e-52<br><br>**Enterotoxin A; enterotoxin-like toxin type P; enterotoxin D; enterotoxin E; Enterotoxin; enterotoxin B.**	Query: 47 - 258 / 260<br>Template: 31 - 236 / 247<br>E-value = 5.4e-52<br><br>**Chain B, Crystal Structure Of Sek; Chain A, Crystal Structure Of Sek; Chain A, Crystal Structure Of Sek-Hvb5.1…**	No homologous regulatory domains found
**SACOL0051**	**Hypothetical protein**	MEFEHKLEKLISEVNNKTEINNYVFFSLGKSSVKAQVKLLKKTNYLKQDISKLALKFKKK<br>SGEFPEWIKLDIVTSTEKILFKELKKTLINTRRNYVDFGIAFDSQWNFAVLPEEINANAF<br>VRPDNTTKELFLSEKNINNYLRKYTTNKKAFSSEFYNEKEVIKFYTQGFFIGDEEVHELY<br>SEGYKKGLRKVNDLNNEIDQLIESSTNFLQNMLLDNGKYIYGYFPHFDNEIGFYNVLRHS<br>SSTYALIEGLSYLGKSLQPVEKAIDYIILNQLFEIGDKAYIFDDTEEANEIKLGQNASFI<br>FAVCEYLKHEDNPKFLESAQKVAKGILSMIDEDTYETTHLLNYPDLSVKEKFRIIYYDGE<br>AALALLRLYQKDENELWLKTVENLMDRFIEKKYWQYHDHWLGYCTNELVQINPQDKYFEF<br>GIKNVNNYLDYIKNRETTFPTFLEMLMATYRLVQKAKDTGREELVNNLIDEQYLIDVINI<br>RADYQRVGFFYPEIAMYFKNPSRILGSFFIKHHGYRVRIDDIEHYLSGYVQYQLAFNR	No structural similarity found				Query: 1 - 538 / 538<br>Template: 8 - 541 / 552<br>E-value = 9.1e-130<br><br>**lmo1798; Poly alpha-glucosyltransferase; glycosyl transferase, group I; lin1912; poly (glycerol-phosphate) alpha-glucosyltransferase.**	Query: 1 - 537 / 538<br>Template: 11 - 546 / 585<br>E-value = 1.9e-128<br><br>**Poly alpha-glucosyltransferase; Mur ligase middle domain protein; mur ligase middle domain-containing protein; Glycosyl Hydrolase Family 88.**	Query: 22 - 537 / 538<br>Template: 60 - 581 / 1067<br>E-value = 5.3e-117<br><br>**Mur ligase middle domain-containing protein; mur ligase middle domain protein.**	Query: 20 - 537 / 538<br>Template: 27 - 549 / 1044<br>E-value = 1.3e-99<br><br>**Poly alpha-glucosyltransferase.**	**26. result**<br>Query: 223 - 404 / 538<br>Template: 431 - 618 / 716<br>E-value = 1.3e-23<br><br>**Six-hairpin glycosidase; transcriptional regulator; glycoside hydrolase family 76; transmembrane protein; gll2144; thymidylate kinase.**<br><br>**94. result**<br>Query: 202 - 385 / 538<br>Template: 379 - 561 / 680<br>E-value = 3.7e-10<br><br>**Transcriptional regulator, Fis family; transmembrane protein.**
**SACOL0052**	**Glycosyl transferase, group 1 family protein; identified by match to protein family HMM PF00534**	MIYSVGNRLGNKLTGIEKAMINRHNLFKNHSITSKLIFASWSPRLHFNASLFQIEDDDIL<br>SLYDVLQDSINIKSIHKDWITHWTKECNYTLKFIENTNDIKIYEKSNYRMYVHFNDSNYQ<br>HLDYINHFDVNQRKIRRDLYDSRGFLSCSRTLTTNQKVVCEHYYTPEGTIKFQKFFNPEL<br>ENSQSQLIIYNTDSQLKYFNNDQELLAFAIEKLYKNGDIFLSDKNINTAPIFNNTSETIP<br>VLAVLHSTHVKNIDMVYESDIKNTYKHVFNNLNRYSGIIVSTKQQQLDISARINNEIPVH<br>TIPVGYIDEHFTNLKRNNHSINNNKIISVARYSPEKQLNHQIELVSKLIKEFPNIQLHLY<br>GFGKEEEKYKQLITEYNLENNVFLRGFRRNLSAEIQDAYMSLITSNMEGFNLGLLETITE<br>GIPPVGYNSKYGPSELILNNENGYLINKNDKDELYNRVRNLLLDKTLRDTFSQECIKHSK<br>AFSSKIVMKLWEDRFSTINSSYE	**SCOP: c.87.1.8**<br>Query: 329 - 483 / 503<br>Template: 201 - 355 / 370<br>E-value = 3e-07<br><br>**Lipopolysaccharide core biosynthesis protein RfaG {Escherichia coli [TaxId: 562]}**	No further structural similarity found			Query: 1 - 499 / 503<br>Template: 3 - 505 / 550<br>E-value = 6.8e-91<br><br>**Glycosyl transferase group 1; glycosyltransferase; lipopolysaccharide biosynthesis protein…**	Query: 1 - 497 / 503<br>Template: 1 - 498 / 511<br>E-value = 7.2e-83<br><br>**Polyglycerol-phosphate alpha-glucosyltransferase; Glycosyl transferase, group 1; poly (glycerol-phosphate) alpha-glucosyltransferase…**	Query: 1 - 499 / 503<br>Template: 15 - 516 / 557<br>E-value = 1.3e-76<br><br>**Poly(Glycerol-phosphate) alpha-glucosyltransferas e; glycosyl transferase; accessory Sec system glycosylation protein GtfA2; glycosyl transferase, group 1 SP1758…**	Query: 1 - 498 / 503<br>Template: 6 - 496 / 504<br>E-value = 3.4e-60<br><br>**Glycosyltransferase; TarM protein; glycosyltransferase, group 1 family protein…**	**212. result**<br>Query: 192 - 502 / 503<br>Template: 75 - 400 / 666<br>E-value = 5.6e-35<br><br>**Bifunctional glycosyl transferase, group 1/ recombination regulator RecX…**
**SACOL0238**	**Teichoic acid biosynthesis protein, putative; identified by match to protein family HMM PF04464**	MTKTKQAIHIDNIYWERVQLYIEGHSEGVDLTSGQFVLRNLTETKTLEANEMKIDGNTFI<br>CRFNVAILDDGYYLPMDKYLFVYHDQLEYIGQLNPNIIDQAYAALNEEQIEEYNELTTQN<br>GKVNYLLAYDAKVFRKGGVSQHTVYTITPEIASDVNEFVFDIEITLPQEKSGVIATSAHW<br>LHKQGHKASFESRSFLFKAIFNITKLLHIKRSKTILFTSDSRPNLSGNFKYVYDELLRQK<br>VDFDYDIKTVFKENITDRRKWRDKFRLPYLLGKADYIFVDDFHPLIYTVRFRPSQEIIQV<br>WHAVGAFKTVGFSRTGKKGGPFIDSLNHRSYTKAYVSSETDIPFYAEAFGIREENVVPTG<br>VPRTDVLFDEAYATQIKQEMEDELPIIKGKKVILFAPTFRGNGHGTAHYPFFKIDFERLA<br>RYCEKHNAVVLFKMHPFVKNRLNISREHRQYFIDVSDHREVNDILFVTDLLISDYSSLIY<br>EYAVFKKPMIFYAFDLEDYITTRDFYEPFESFVPGKIVQSFDALMDALDNEDYEVEKVVP<br>FLDKHFKYQDGRSSERLVKDLFRR	No structural similarity found				Query: 1 - 564 / 564<br>Template: 1 - 562 / 565<br>E-value = 2.2e-125<br><br>**Glycosyl/ glycerophosphate transferase; CDP-glycerol: poly(glycerophosphate) glycerophospho-transferase; teichoic acid biosynthesis protein B…**	Query: 1 - 372 / 564<br>Template: 3 - 374 / 374<br>E-value = 5.5e-88<br><br>**Teichoic acid biosynthesis protein B; teichoic acid biosynthesis family protein.**	Query: 8 - 561 / 564<br>Template: 17 - 549 / 558<br>E-value = 4.5e-78<br><br>**CDP-glycerol:poly-glycerophosphate transferase; glycosyl transferase, group 2 family.**	Query: 1 - 270 / 564<br>Template: 1 - 270 / 270<br>E-value = 2.1e-75<br><br>**Teichoic acid biosynthesis protein, putative.**	**232. result**<br>Query: 290 - 547 / 564<br>Template: 112 - 371 / 666<br>E-value = 3.7e-17<br><br>**Bifunctional glycosyl transferase, group 1/ recombination regulator RecX…**<br><br>**291. result**<br>Query: 322 - 558 / 564<br>Template: 116 - 336 / 360<br>E-value = 6.9e-16<br><br>**Fis family transcriptional regulator; glycosyl transferase…**
**SACOL0239**	**TagF domain-containing protein; identified by match to protein family HMM PF04464**	MIKNTIKKLIEHSIYTTFKLLSKLPNKNLIYFESFHGKQYSDNPKALYEYLTEHSDAQLI<br>WGVKKGYEHIFQQHNVPYVTKFSMKWFLAMPRAKAWMINTRTPDWLYKSPRTTYLQTWHG<br>TPLKKIGLDISNVKMLGTNTQNYQDGFKKESQRWDYLVSPNPYSTSIFQNAFHVSRDKIL<br>ETGYPRNDKLSHKRNDTEYINGIKTRLNIPLDKKVIMYAPTWRDDEAIREGSYQFNVNFD<br>IEALRQALDDDYVILLRMHYLVVTRIDEHDDFVKDVSDYEDISDLYLISDALVTDYSSVM<br>FDFGVLKRPQIFYAYDLDKYGDELRGFYMDYKKELPGPIVENQTALIDALKQIDETANEY<br>IEARTVFYQKFCSLEDGQASQRICQTIFK	No structural similarity found				Query: 1 - 389 / 389<br>Template: 53 - 433 / 441<br>E-value = 1.9e-57<br><br>**Teichoic acid biosynthesis protein F; TagF protein; TagF domain-containing protein; glycosyl transferase…**	Query: 13 - 389 / 389<br>Template: 16 - 382 / 396<br>E-value = 1.3e-53<br><br>**teichoic acid biosynthesis protein RodC related protein; CDP-Glycerol:Poly(glycero-phosphate) glycerophospho-transferase family…**	Query: 21 - 389 / 389<br>Template: 387 - 753 / 759<br>E-value = 9.2e-53<br><br>**TagF domain protein; teichoic acid biosynthesis protein; teichoic acid biosynthesis protein F.**	Query: 7 - 389 / 389<br>Template: 334 - 718 / 720<br>E-value = 1e-48<br><br>**Polyglycerol phosphate polymerase; Major teichoic acid biosynthesis protein F; CGPTase; CDP-glycerol:poly(glycero-phosphate) glycerophosphotransferase.**	**133. result**<br>Query: 150 - 360 / 389<br>Template: 121 - 316 / 360<br>E-value = 2.5e-33<br><br>**Fis family transcriptional regulator; glycosyl transferase…**<br><br>**152. result**<br>Query: 152 - 357 / 389<br>Template: 159 - 358 / 666<br>E-value = 2.3e-32<br><br>**Bifunctional glycosyl transferase, group 1/ recombination regulator RecX…**
**SACOL0694**	**Teichoic acids export protein ATP-binding subunit; with TagG is involved in the export of teichoic acids**	MNVSVNIKNVTKEYRIYRTNKERMKDALIPKHKNKTFFALDDISLKAYEGDVIGLVGING<br>SGKSTLSNIIGGSLSPTVGKVDRNGEVSVIAISAGLSGQLTGIENIEFKMLCMGFKRKEI<br>KAMTPKIIEFSELGEFIYQPVKKYSSGMRAKLGFSINITVNPDILVIDEALSVGDQTFAQ<br>KCLDKIYEFKEQNKTIFFVSHNLGQVRQFCTKIAWIEGGKLKDYGELDDVLPKYEAFLND<br>FKKKSKAEQKEFRNKLDESRFVIK	**SCOP: c.37.1.12**<br>Query: 35 - 221 / 264<br>Template: 10 - 211 / 238<br>E-value = 2e-20<br><br>**Putative ABC transporter TM0544 {Thermotoga maritima [TaxId: 2336]}**	**SCOP: c.37.1.12**<br>Query: 37 - 220 / 264<br>Template: 14 - 199 / 200<br>E-value = 2e-20<br><br>**Putative ABC transporter PF0895 {Pyrococcus furiosus [TaxId: 2261]}**	**SCOP: c.37.1.12**<br>Query: 7 - 230 / 264<br>Template: 4 - 228 / 240<br>E-value = 2e-16<br><br>**Methionine import ATP-binding protein MetN {Escherichia coli [TaxId: 562]}**	**SCOP: c.37.1.12**<br>Query: 40 - 238 / 264<br>Template: 18 - 249 / 258<br>E-value = 3e-15<br><br>**ATP-binding subunit of the histidine permease {Salmonella typhimurium [TaxId: 90371]}**	Query: 1 - 264 / 264<br>Template: 21 - 285 / 336<br>E-value = 2.7e-106<br><br>**Teichoic acids export ATP-binding protein TagH.**	Query: 1 - 259 / 264<br>Template: 5 - 263 / 555<br>E-value = 2.4e-94<br><br>**Polysaccharide ABC transporter, ATP-binding protein; polysaccharide ABC transporter ATP-binding protein; teichoic acid export ATP-binding protein…**	Query: 2 - 260 / 264<br>Template: 4 - 263 / 336<br>E-value = 2.4e-85<br><br>**ABC transporter ATP-binding protein; ABC transporter, ATP-binding protein; teichoic acids export ATP-binding protein TagH…**	Query: 2 - 260 / 264<br>Template: 6 - 265 / 435<br>E-value = 4.6e-83<br><br>**Teichoic acid ABC superfamily ATP binding cassette transporter, ABC protein; teichoic acid ABC transporter; teichoic acid transport system ATP-binding protein…**	**135. result**<br>Query: 1 - 234 / 264<br>Template: 1 - 231 / 249<br>E-value = 1.3e-37<br><br>**Macrolide export ATP-binding/permease protein MacB; ABC transporter family protein; glutamine ABC transporter ATP-binding protein; cystic fibrosis transmembrane conductance regulator…**
**SACOL0695**	**TagG protein, teichoic acid ABC transporter protein, putative; identified by similarity to EGAD:30975; match to protein family HMM PF01061**	MSAIGTVFKEHVKNFYLIQRLAQFQVKIINHSNYLGVAWELINPVMQIMVYWMVFGLGIR<br>SNAPIHGVPFVYWLLVGISMWFFINQGILEGTKAITQKFNQVSKMNFPLSIIPTYIVTSR<br>FYGHLGLLLLVIIACMFTGIYPSIHIIQLLIYVPFCFFLTASVTLLTSTLGVLVRDTQML<br>MQAILRILFYFSPILWLPKNHGISGLIHEMMKYNPVYFIAESYRAAILYHEWYFMDHWKL<br>MLYNFGIVAIFFAIGAYLHMKYRDQFADFL	No structural similarity found				Query: 2 - 270 / 270<br>Template: 26 - 291 / 292<br>E-value = 4e-38<br><br>**ABC type transport system integral membrane protein; ABC-2 type transport system integral membrane protein…**	Query: 5 - 270 / 270<br>Template: 42 - 298 / 298<br>E-value = 1.7e-37<br><br>**ABC transporter permease protein; permease protein of ABC transporter; lipopolysaccharide transport system permease…**	Query: 5 - 270 / 270<br>Template: 44 - 302 / 304<br>E-value = 2.7e-37<br><br>**ABC-2 type transporter superfamily; polysaccharide/polyol phosphate ABC transporter permease…**	Query: 4 - 270 / 270<br>Template: 21 - 283 / 284<br>E-value = 8.4e-37<br><br>**Lipopolysaccharide ABC transporter, permease protein; lipopolysaccharide ABC transporter permease; O-antigen export system permease protein rfbA…**	No homologous regulatory domains found
**SACOL0936**	**DltB protein; identified by similarity to EGAD:22430; match to protein family HMM PF03062**	MIPYGDFTFFLIALIALLPVIILGFLGKRSYIYNGVVTAFMIVLIFSSDKHNLFDQKYLS<br>VQLISFIIYVVWQVLLIMFYYHSKPKNNSFSKFVTVMVLSILPLALVKVLQSTWLGGHQI<br>HFHESKLIEFVGFLGISYVTFKSVQLIMEIRDGSIKEIKVWKLIQFISFFPTISSGPIDR<br>YKRFVKDDKKVPTGNEYRELVLKAIHMIMLGFLYKYIVAYFINTYAIMPLQLDLHGFVNL<br>WLYMYAYSLYLFFDFAGYSLFAIAFSYLFGIKTPPNFDKPFKAKNIKDFWNRWHMTLSFW<br>FRDCIYMRSLFYMSRKKLLKSQFAMSNVAFLINFFIMGIWHGIEVYYIVYGLYHAALFIG<br>YGYYERWRKKHPPRWQNGFTTALSIVITFHFVTFGFLIFSGKLI	No structural similarity found				Query: 15 - 399 / 404<br>Template: 29 - 389 / 481<br>E-value = 3.6e-103<br><br>**Alginate O-acetyltransferase AlgI; alginate O-acetylation protein (AlgI)…**	Query: 14 - 399 / 404<br>Template: 35 - 399 / 505<br>E-value = 1.4e-102<br><br>**Alginate O-acetyltransferase AlgI; AlgI; alginate O-acetylation protein; alginate O-acetylation protein AlgI; cellulose acetylase…**	Query: 14 - 399 / 404<br>Template: 19 - 382 / 456<br>E-value = 3.4e-102<br><br>**Alginate O-acetylation protein AlgI; Membrane bound O-acyl transferase MBOAT…**	Query: 15 - 400 / 404<br>Template: 10 - 372 / 466<br>E-value = 7e-102<br><br>**Probable poly(beta-D-mannuronate) O-acetylase.**	No homologous regulatory domains found
**SACOL0938**	**DltD protein; identified by similarity to EGAD:16580; match to protein family HMM PF04914; match to protein family HMM PF04915; match to protein family HMM PF04918**	MKLKPFLPILISGAVFIVFLLLPASWFTGLVNEKTVEDNRTSLTDQVLKGTLIQDKLYES<br>NKYYPIYGSSELGKDDPFNPAIALNKHNANKKAFLLGAGGSTDLINAIELASQYDKLKGK<br>KLTFIISPQWFTNHGLTNQNFDARMSQTQINQMFQQKNMSTELKRRYAQRLLQFPHVHNK<br>EYLKSYAKNPKETKDSYISGFKENQLIKIEAIKSLFAMDKSPLEHVKPATKPDASWDEMK<br>QKAVEIGKADTTSNKFGIRDQYWKLIQESKRKVRRDYEFNVNSPEFQDLELLVKTMRAAG<br>ADVQYVSIPSNGVWYDHIGIDKERRQAVYKKIHSTVVDNGGKIYDMTDKDYEKYVISDAV<br>HIGWKGWVYMDEQIAKHMKGEPQPEVDKPKN	No structural similarity found				Query: 6 - 381 / 391<br>Template: 17 - 400 / 479<br>E-value = 3.5e-133<br><br>**Chain F, Crystal Structure Of D-Alanyl-Lipoteichoic Acid Synthetase From Streptococcus Pneumoniae R6…**	Query: 1 - 384 / 391<br>Template: 1 - 394 / 407<br>E-value = 4.7e-121<br><br>**Flags: Precursor.**	Query: 3 - 381 / 391<br>Template: 4 - 393 / 420<br>E-value = 1.4e-106<br><br>**D-alanyl-lipoteichoic acid biosynthesis protein DltD; DltD central region; DltD domain protein; DltD C; D-alanine transferase DltD; D-alanine transferase.**	Query: 6 - 381 / 391<br>Template: 11 - 396 / 777<br>E-value = 9.1e-99<br><br>**D-alanyl-lipoteichoic acid biosynthesis protein DltD; DltD precursor; beta-lactamase.**	No homologous regulatory domains found
