# Supplementary material for: A Staphylococcus aureus Proteome Overview: Shared and Specific Proteins and Protein Complexes from Representative Strains of All Three Clades
Source: Proteomes. 2016 Feb 19;4(1):8. doi: 10.3390/proteomes4010008 (PMC5217359; doi:10.3390/proteomes4010008)
Supplement: Supplementary file 1 [file proteomes-04-00008-s001.zip › proteomes-04-00008-supplementary/Supplementary File S4-Table S4-examplesComplexModification layout.pdf]

proteomes

MDPI

Table S4. Examples for how Protein complexes may be modified (time, post-translational, ribonucleoproteins). (**a**) Heterogeneous complexes: Ribonucleoproteins; (**b**) Changes over time and in metabolic adaptation. There is variation in protein complexes during time and different modes regulate this.

(a)

–RNAse P *in S. aureus:* It removes the leader pre-tRNA sequence. It forms a complex with RNAseP protein. In *S. aureus* even the 3D structure of the RNA P holoenzyme with its single-stranded RNA is known (1D6T; [1])
–*tmRNA in S. aureus:* Ssra/tmRNA, base pairing to crtMN mRNA, but having an interaction (regulation of translation) with proteins leading to carotenoid pigment *crtM* and *crtN* genes, a key feature of pathogenic S. aureus [2]; furthermore, there is also a RNA-protein interaction involved: SmpB (small protein B) can bind *ssrA* and is involved in ssrA biological function in *Escherichia coli;* in *S. aurues* this is present according to Rfam prediction (RFAM family RF00023).
–summary number on sRNAs: thirty are well known and involved in translational but also protein interactions including central regulation of metabolism RsaE [3] to coordinate metabolism when carbon sources get low. New sRNAs have been discovered recently, for instance ArtR [4], which regulates a-toxin expression via the untranslated region of the sarT mRNA.

(b)

Example 1: The diauxic shift leads to a reassembly of aldolase and pyruvate dehydrogenase complexes as well as changes the activities of the transaminases, amino acid synthesis complexes etc.
Example 2: Redox changes affect protein complexes via Rex-family of repressors [5]. This has then impact on numerous protein complexes, in particular global protein synthesis and on the activity of fermentation pathways under aerobic and anaerobic conditions.
Example 3: Anaerobic conditions The *Staphylococcus aureus* SrrAB two-component system promotes resistance to nitrosative stress and hypoxia. [6].
**A general repository:** Much more data on these condition-specific proteome changes (without looking at individual protein complexes as analyzed here) can be found in Aureolib [7]. In infection biology of course protection against *S. aureus* is important, we analyze for this the human monoclonal antibody targeting the conserved staphylococcal antigen IsaA (cleaving peptidoglycan) protects mice against *Staphylococcus aureus* bacteremia [8]. Interestingly, the IsaA protein is present in all five strains and has a key interaction with SACOL2583 acetyltransferase. Further predicted interactions include a suitable transporter and cell wall protein ScdA. In particular the latter could boost a potential vaccine further.

References

1. Spitzfaden, C.; Nicholson, N.; Jones, J.J., Guth, S.; Lehr, R.; Prescott, C.D.; Hegg, L.A.; Eggleston, D.S. The structure of ribonuclease P protein from *Staphylococcus aureus* reveals a unique binding site for single-stranded RNA. *J. Mol. Biol.* **2000**, *295*, 105–115.
2. Liu, Y.; Wu, N.; Dong, J.; Gao, Y.; Zhang, X.; Shao, N.; Yang, G. SsrA (tmRNA) acts as an antisense RNA to regulate *Staphylococcus aureus* pigment synthesis by base pairing with crtMN mRNA. *FEBS Lett.* **2010**, *584*, 4325–4329.
3. Bohn, C.; Rigoulay, C.; Chabelskaya, S.; Sharma, C.M.; Marchais, A.; Skorski, P.; Borezée-Durant, E.; Barbet, R.; Jacquet, E.; Jacq, A.; *et al*. Experimental discovery of small RNAs in *Staphylococcus aureus* reveals a riboregulator of central metabolism. *Nucleic Acids Res.* **2010**, *38*, 6620–6636.
4. Xue, T.; Zhang, X.; Sun, H.; Sun, B. ArtR, a novel sRNA of *Staphylococcus aureus*, regulates α-toxin expression by targeting the 5′ UTR of sarT mRNA. *Med. Microbiol. Immunol.* **2014**, *203*, 1–12.
5. Pagels, M.; Fuchs, S.; Pané-Farré, J.; Kohler, C.; Menschner, L.; Hecker, M.; McNamarra, P.J.; Bauer, M.C.; von Wachenfeldt, C.; Liebeke, M.; *et al*. Redox sensing by a Rex-family repressor is involved in the regulation of anaerobic gene expression in *Staphylococcus aureus*. *Mol. Microbiol.* **2010**, *76*, 1142–1161.
6. Kinkel, T.L.; Roux, C.M.; Dunman, P.M.; Fang, F.C. The *Staphylococcus aureus* SrrAB two-component system promotes resistance to nitrosative stress and hypoxia. *mBio* **2013**, *4*, doi:10.1128/mBio.00696-13.

7. Fuchs, S.; Zühlke, D.; Pané-Farré, J.; Kusch, H.; Wolf, C.; Reiß, S.; le Binh, T.N.; Albrecht, D.; Riedel, K.; Hecker, M.; *et al.*, Aureolib—A proteome signature library: Towards an understanding of *staphylococcus aureus* pathophysiology. *PLoS ONE* **2013**, *8*, e70669.
8. Van den Berg, S.; Bonarius, H.P.; van Kessel, K.P.; Elsinga, G.S.; Kooi, N.; Westra, H.; Bosma, T., van der Kooi-Pol, M.M.; Koedijk, D.G.; Groen, H.; *et al*. A human monoclonal antibody targeting the conserved staphylococcal antigen IsaA protects mice against *Staphylococcus aureus* bacteremia. *Int. J. Med. Microbiol.* **2015**, *305*, 55–64.

© 2016 by the authors; licensee MDPI, Basel, Switzerland. This article is an open access article distributed under the terms and conditions of the Creative Commons by Attribution (CC-BY) license (http://creativecommons.org/licenses/by/4.0/).
